# Gene regulation by convergent promoters

**DOI:** 10.1038/s41588-024-02025-w

**Published:** 2025-01-06

**Authors:** Elina Wiechens, Flavia Vigliotti, Kanstantsin Siniuk, Robert Schwarz, Katjana Schwab, Konstantin Riege, Alena van Bömmel, Ivonne Görlich, Martin Bens, Arne Sahm, Marco Groth, Morgan A. Sammons, Alexander Loewer, Steve Hoffmann, Martin Fischer

**Affiliations:** 1 Hoffmann Lab, Leibniz Institute on Aging–Fritz Lipmann Institute (FLI), Jena, Germany; 2 Department of Biology, Systems Biology of the Stress Response, Technical University of Darmstadt, Darmstadt, Germany; 3 Core Facility Next Generation Sequencing, Leibniz Institute on Aging–Fritz Lipmann Institute (FLI), Jena, Germany; 4 Computational Phenomics Group, IUF–Leibniz Research Institute for Environmental Medicine, Düsseldorf, Germany; 5 Computational Phenomics Group, Ruhr University Bochum, Bochum, Germany; 6 Department of Biological Sciences, The RNA Institute, The State University of New York at Albany, Albany, NY USA

**Keywords:** Gene regulation, Epigenomics, Transcriptomics, Data processing

## Abstract

Convergent transcription, that is, the collision of sense and antisense transcription, is ubiquitous in mammalian genomes and believed to diminish RNA expression. Recently, antisense transcription downstream of promoters was found to be surprisingly prevalent. However, functional characteristics of affected promoters are poorly investigated. Here we show that convergent transcription marks an unexpected positively co-regulated promoter constellation. By assessing transcriptional dynamic systems, we identified co-regulated constituent promoters connected through a distinct chromatin structure. Within these *cis*-regulatory domains, transcription factors can regulate both constituting promoters by binding to only one of them. Convergent promoters comprise about a quarter of all active transcript start sites and initiate 5′-overlapping antisense RNAs—an RNA class believed previously to be rare. Visualization of nascent RNA molecules reveals convergent cotranscription at these loci. Together, our results demonstrate that co-regulated convergent promoters substantially expand the *cis*-regulatory repertoire, reveal limitations of the transcription interference model and call for adjusting the promoter concept.

## Abstract (web summary)

Genome-wide analysis and genetic manipulation at loci regulated by p53, E2F4 and RFX7 show that convergent promoters with similar epigenetic features can be co-regulated and simultaneously expressed in the same direction.

## Main

**Fig. 1:**
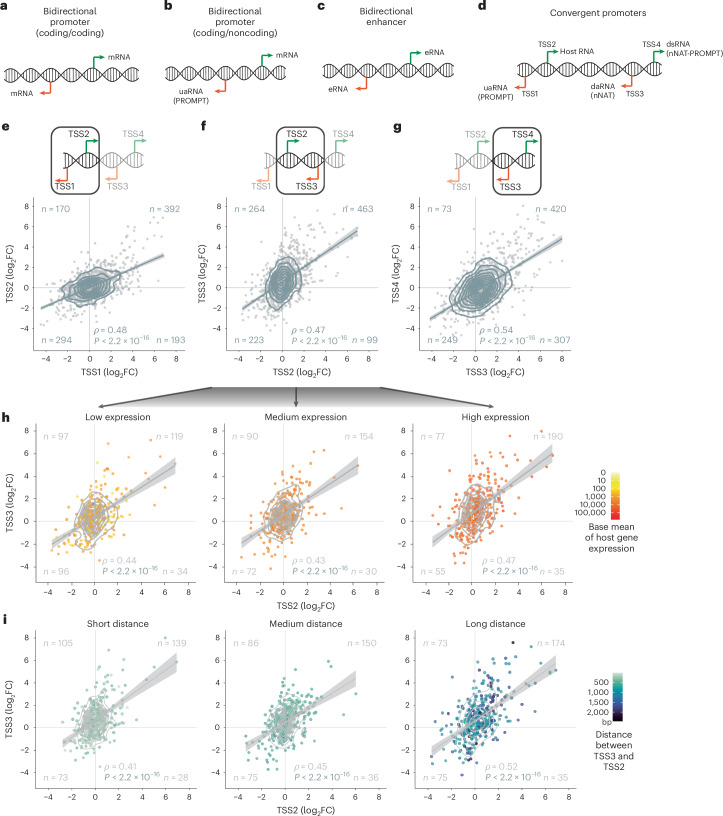
Convergent promoter transcription is positively correlated. **a**–**d**, Schematics of TSSs and RNA classes at bidirectional promoters that initiate two coding genes (**a**) or a coding gene and a noncoding RNA (**b**), bidirectional enhancers (**c**) and convergent promoters (**d**). **e**–**g**, Expression dynamics (log_2_FC Nutlin-3a compared with DMSO control) between the divergent TSSs TSS1 and TSS2 (**e**) as well as TSS3 and TSS4 (**f**) and the convergent TSSs, TSS2 and TSS3 (**g**). Schematics (top panels) highlight the TSSs that have been compared for their log_2_FC correlation (bottom panels). **h**, The convergent TSSs, TSS2 and TSS3, separated into three expression groups (low, medium and high) based on the base mean expression of host gene expression (elicited by TSS2). **i**, The convergent TSSs, TSS2 and TSS3, have been separated into three groups based on the distance between TSS2 and TSS3. **e**–**i**, Data from MCF-7 cells. Linear regression with 95% confidence intervals. Spearman correlation with two-tailed significance. Source data

Transcriptional coordination by enhancers and promoters is a fundamental process central to development and disease^1–3^. Both promoters and enhancers provide DNA regions that transcription factors can bind to regulate transcription, but only promoters initiate the transcription of genes. Over the past decade, active enhancers and promoters have been shown to initiate divergent, bidirectional RNA transcription^4–6^. These include bidirectional promoters generating divergent messenger RNAs (mRNAs)^7,8^ (Fig. 1a), as well as promoters producing upstream antisense RNAs (uaRNAS), a type of noncoding RNA (ncRNA), in addition to the sense mRNA^9–11^. The latter are also called promoter upstream transcripts (PROMPTs) (Fig. 1b). Enhancers can also initiate bidirectional transcription of two divergent enhancer RNAs (eRNAs)^4^ (Fig. 1c).

Although it has long been known that overlapping antisense transcripts are abundant^12–16^, it has been discovered only recently that antisense transcription downstream of promoters is surprisingly prevalent. In such configurations, antisense transcription is driven by two juxtaposed promoters, each of which drives divergent transcription^17–19^. These characteristics yield a complex constellation of four proximal TSSs that produce (1) a uaRNA (PROMPT) through the first antisense TSS (TSS1), (2) the host RNA through the first sense TSS (TSS2), (3) a downstream antisense RNA (daRNA; also known as nNAT (novel natural antisense transcript)) from the second antisense TSS (TSS3) and (4) a downstream sense RNA (dsRNA; also known as nNAT-PROMPT) from the second sense TSS (TSS4)^18^ (Fig. 1d). The host RNA and the daRNA are convergently transcribed within this quadruple. We refer to all pairs of juxtaposed promoters that elicit convergent transcription between each other as ‘convergent promoters.’ However, it was unclear whether convergent promoters are functionally different from other promoters.

Convergent transcription is generally believed to evoke transcription interference^20–25^. In agreement with this model, the expression of daRNAs has been reported to be correlated negatively with host RNA expression^17,18,26^. However, one study found no correlation between the expression of daRNA and host mRNA^19^. Whereas several antisense transcripts inhibit gene expression^14,27–32^, antisense transcripts have also been shown to promote gene expression^14,33–36^. Thus, the regulatory relationship between sense and antisense transcription appears to transcend the simple concepts of inhibition or activation.

## Results

### Convergent promoter transcription correlates positively

Given that previous studies have investigated convergent transcription at promoters in a single cell condition^17–19,26^, we wondered whether a dynamic experimental setup would provide more detailed insight into their functionality. To this end, we treated cells with the MDM2 inhibitor Nutlin-3a—a specific inducer of the transcription factor p53 (ref. ^37^). Activation of p53 causes both up and downregulation of hundreds of RNAs, with different transcription factors involved in each response^38,39^. The specific and well-characterized effects of Nutlin-3a treatment provide a robust framework for studying convergent transcription at promoters and its dynamics. We employed three widely used cell systems: the breast cancer cell line MCF-7, the osteosarcoma cell line U2OS, and the hTERT-immortalized noncancerous retina-pigmented epithelium cell line RPE-1—all of which possess wild-type p53. To accurately capture TSSs, we utilized cap analysis gene expression and deep sequencing (CAGE–seq) (average of ~92 million reads per condition) of the three cell lines under Nutlin-3a and dimethylsulfoxide (DMSO) solvent control treatment conditions (Methods).

**Extended Data Fig. 1:**
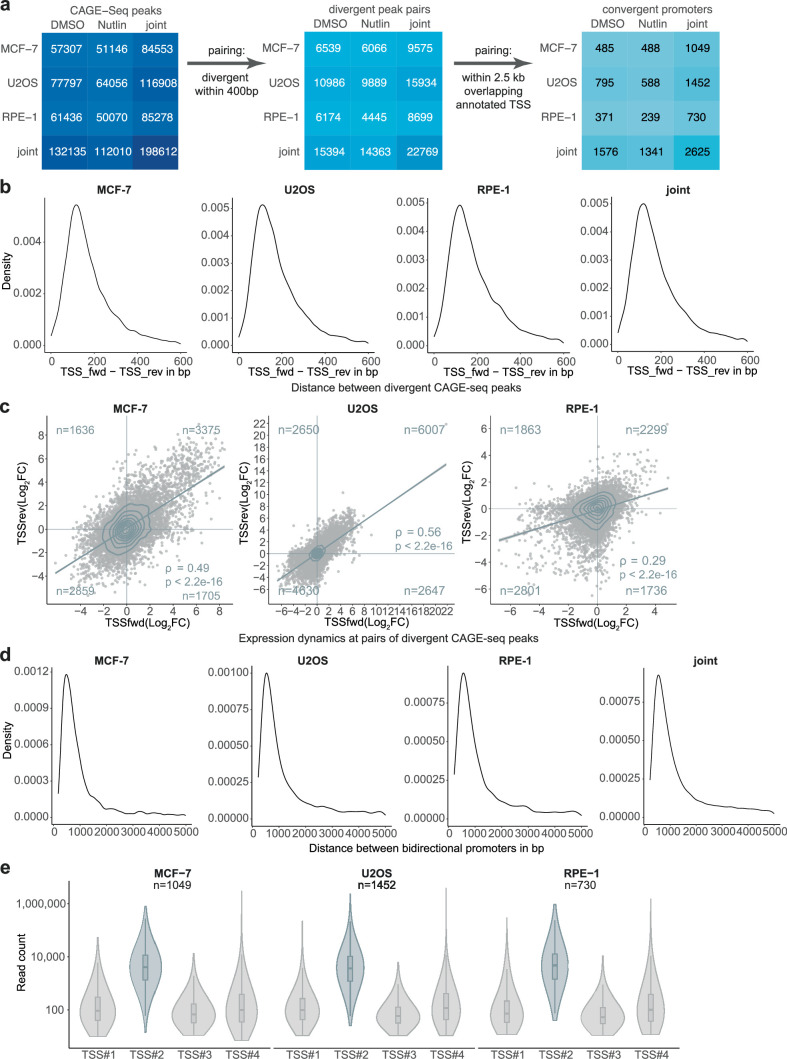
Identification of convergent promoters. **(a)** Workflow of convergent promoter identification using CAGE–seq peaks and heatmaps displaying the respective numbers. **(b)** Profile of the distance between divergent (*-strand* followed by *+strand*) CAGE-seq peaks supports the established threshold of 400 bp for pairing. **(c)** Expression dynamics (Log2FC Nutlin-3a vs DMSO control treatment) at pairs of divergent CAGE-seq peaks display a positive expression. Spearman correlation with two-tailed significance. **(d)** Profile of the distance between pairs of divergent CAGE-seq peaks (promoters) indicates 2500 bp as a suitable threshold for pairing promoters to convergent promoters. **(e)** Violin plots summarize the read-count values of the four TSSs of the convergent promoters identified in the respective cell lines. Boxes show the median, upper, and lower quartiles, whiskers 1.5x interquartile range. Source data

**Extended Data Fig. 2:**
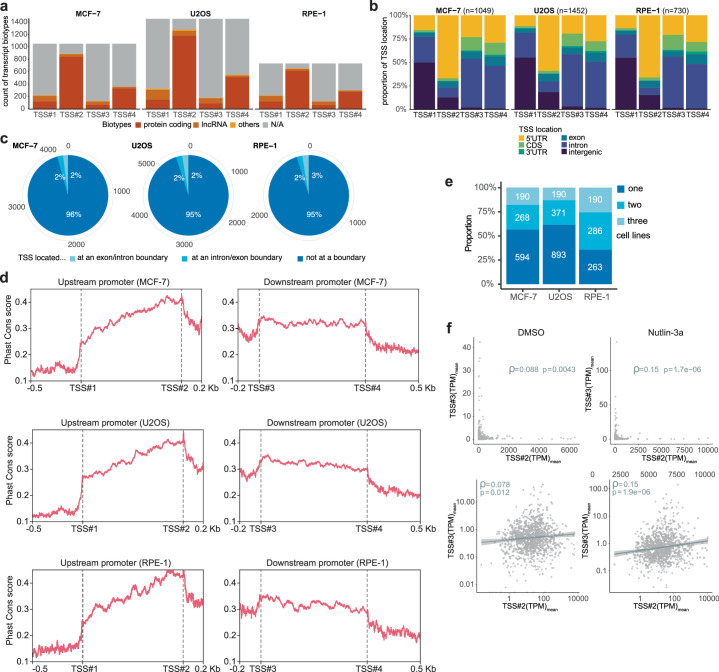
Characteristics of convergent promoters and their TSSs. **(a)** Biotypes associated with GENCODE-annotated TSSs that overlap with the CAGE-seq peaks harboring the respective convergent promoter TSSs. Many CAGE-seq peaks overlapped with no GENCODE-annotated TSS (N/A – not available). **(b)** Genomic location of convergent promoter TSSs. **(c)** Convergent promoter TSSs located within 5 bp from exon/intron and intron/exon boundaries. **(d)** Summary profiles of PhastCons conservation scores at convergent promoters. **(e)** Convergent promoters were identified using CAGE-seq data from the respective cell lines, and the proportion determined in one, two, or all three cell lines is displayed. **(f)** Baseline expression (transcripts per million, TPM) of the detected TSSs. Linear regression with 95% confidence intervals. Spearman correlation with two-tailed significance. Source data

The number of CAGE–seq peaks detected was similar across conditions and cell lines. The union of all data across conditions and cell lines was used to detect convergent promoters with higher power (Extended Data Fig. 1a). Next, we paired divergent CAGE–seq peaks, that is, all −strand peaks followed by a +strand peak, using an established threshold of 400 bp (ref. ^40^) to predict bidirectional promoters (Extended Data Figs. 1a,b). As expected, we observed a positive correlation of the fold changes of divergent transcripts in all three cell lines comparing Nutlin-3a with DMSO control. (Extended Data Fig. 1c). To predict convergent promoters, that is, juxtaposed promoters with convergent transcription, we intersected pairs of divergent peak pairs and annotated GENCODE TSSs. The most highly expressed TSS was labeled TSS2 (host gene TSS) for orientation purposes (Extended Data Fig. 1d,e and Supplementary Tables 1–4) and coincided largely with annotated protein-coding genes (Extended Data Fig. 2a). TSS2 was predominantly located in 5′ UTRs, indicating that it often represents the canonical TSS of a gene.

In contrast, TSS1 was located predominantly in intergenic regions, whereas TSS3 and TSS4 were located in intronic regions (Extended Data Fig. 2b). Given its location within the host gene and on the host gene strand, TSS4 could serve as an alternative start site for the host gene. Although many TSS4s do indeed overlap known alternative start sites, most do not (Extended Data Fig. 2a). Less than 5% of TSSs in the convergent promoter constellations we identified were located at exon–intron or intron–exon junctions (Extended Data Fig. 2c), suggesting that capped small RNAs, which may be generated upon splicing events^41^, had little or no effect on our detection approach. Conservation data indicate that both promoters, that is, the regions between TSS1 and TSS2 and between TSS3 and TSS4, are more conserved than their surrounding DNA content (Extended Data Fig. 2d), providing critical evidence that both regions are under selection and may have relevant *cis*-regulatory potential. Notably, the average conservation of the region between TSS3 and TSS4 exceeds that of the downstream sequences, which often contain UTRs and coding regions (Extended Data Fig. 2b). Many convergent promoters were identified in several cell lines (Extended Data Fig. 2e), suggesting a more global relevance.

**Extended Data Fig. 3:**
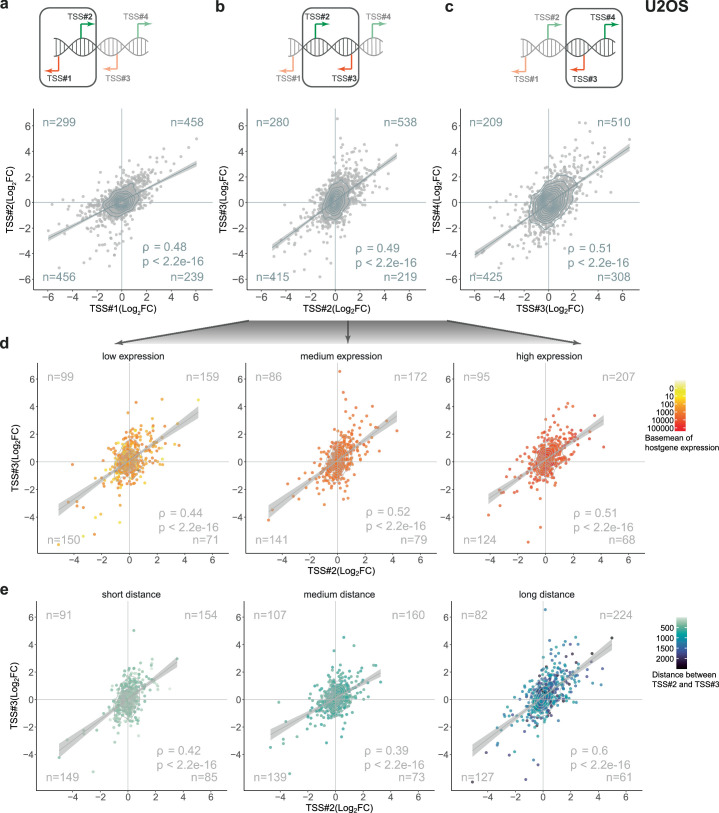
Convergent promoter TSSs display a positive correlation. The expression dynamics (Log2FC Nutlin-3a compared to DMSO control) between the divergent TSSs **(a)** #1 and #2 as well as **(b)** #3 and #4 and the **(c)** convergent TSSs #2 and #3. **(a-c)** Schematics (top panel) highlight the TSSs that have been compared for their log2FC correlation (bottom panel). **(d)** The convergent TSSs #2 and #3 have been separated into three groups based on the basemean expression of host gene expression (elicited by TSS#2). **(e)** The convergent TSSs #2 and #3 have been separated into three groups based on the distance between TSS#2 and #3. **(a-e)** Data from U2OS cells. Linear regression with 95% confidence intervals. Spearman correlation with two-tailed significance. Source data

**Extended Data Fig. 4:**
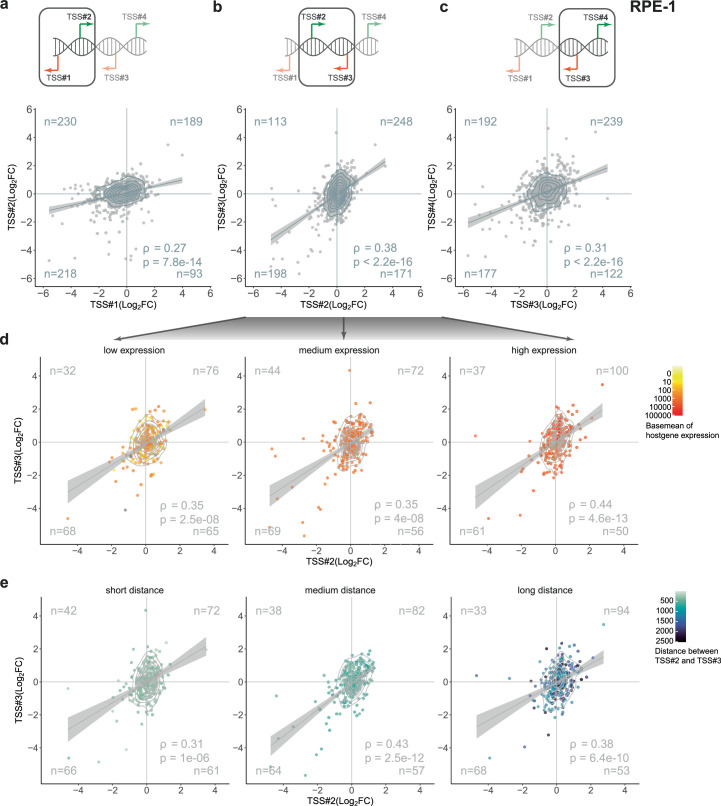
Convergent promoter TSSs display a positive correlation. The expression dynamics (Log2FC Nutlin-3a compared to DMSO control) between the divergent TSSs **(a)** #1 and #2 as well as **(c)** #3 and #4 and the **(b)** convergent TSSs #2 and #3. **(a-c)** Schematics (top panel) highlight the TSSs that have been compared for their log2FC correlation (bottom panel). **(d)** The convergent TSSs #2 and #3 have been separated into three groups based on the basemean expression of host gene expression (elicited by TSS#2). **(e)** The convergent TSSs #2 and #3 have been separated into three groups based on the distance between TSS#2 and #3. **(a-e)** Data from RPE-1 cells. Linear regression with 95% confidence intervals. Spearman correlation with two-tailed significance. Source data

**Extended Data Fig. 5:**
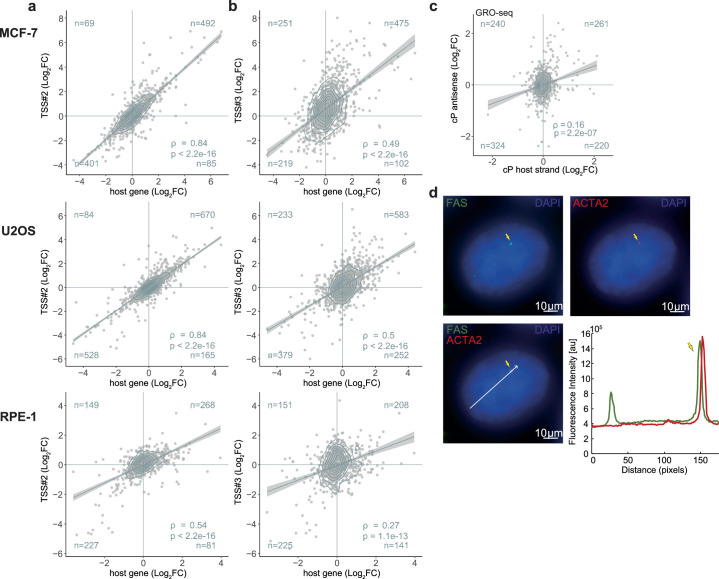
CAGE-seq data validation and additional GRO-seq and smFISH data. **(a)** Differential expression was measured by CAGE-seq (y-axis) compared to differential expression, which was measured by RNA-seq (x-axis) for the host genes (initiated at TSS#2). **(b)** Differential host gene expression was measured by RNA-seq (x-axis) compared to differential expression from the overlapping antisense TSS#3 measured by CAGE-seq (y-axis). **(a,b)** Data from MCF-7 (top panel), U2OS (middle panel), and RPE-1 cells (bottom panel). **(c)** Differential GRO-seq data from Nutlin-3a and DMSO control-treated MCF-7 cells (GSE86165) at convergent promoter regions’ sense and antisense strand. **(a-c)** Linear regression with 95% confidence intervals. Spearman correlation with two-tailed significance. **(d)** Complementary to Fig. 2c. The first intronic sequences of *FAS* and *ACTA2* have been dual color labeled using smFISH. Microscopy images (top panels) display expression of nascent *FAS* (green, left image), *ACTA2* (red, middle image), and their overlap (right image) in Nutlin-3a-treated MCF-7 cells. The fluorescent intensity profiles at the region of interest (white line/arrow in the right image) highlight the overlap of nascent *FAS* and *ACTA2* expression (right panel), which provides evidence for their co-transcription from the same locus. Source data

Unexpectedly, the dynamics of TSS activity within convergent promoters revealed a significant positive correlation not only for those initiating divergent transcription but also for those initiating convergent host RNA and daRNA transcription (Fig. 1e–g and Extended Data Figs. 3a–c and 4a–c). The proportion of convergent TSSs with a negative correlation was essentially the same as for divergent TSSs, where co-regulation is expected. In contrast to the dynamic expression data, the baseline expression between the convergent TSS2 and TSS3 showed only a small, albeit significantly positive, correlation (Extended Data Fig. 2f). For CAGE–seq data validation, we compared them to RNA sequencing (RNA-seq) data. The differential expression of the TSS2 measured by CAGE–seq showed a strong positive correlation with the differential expression of the host gene measured by RNA-seq (Extended Data Fig. 5a). Similar to the positive correlation between the convergent TSS2 and TSS3, the data show a positive correlation also between host gene expression and TSS3 expression (Extended Data Fig. 5b).

**Extended Data Fig. 6:**
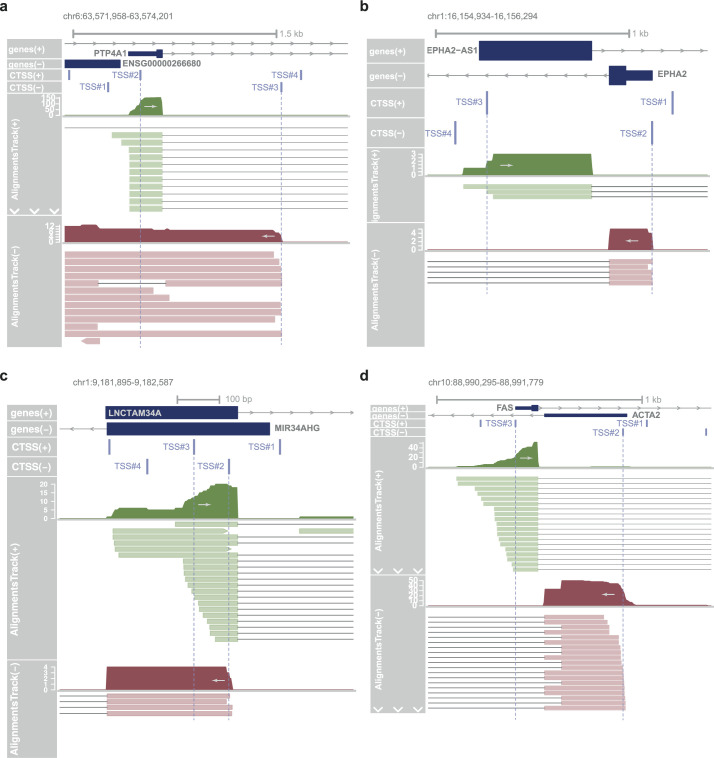
Convergent promoter transcription elongates over each other. Nanopore long-reads from Nutlin-3a-treated MCF-7 cells at the convergent promoter loci of **(a)** *PTP4A1*, **(b)** *EPHA2*/*EPHA2-AS1*, **(c)** *MIR34AHG*/*LNCTAM34A*, and **(d)** *FAS/ACTA2*.

We generated and examined Nanopore long-reads to test whether transcription from TSS2 traverses TSS3 and vice versa. The sequencing data show that convergent transcription between convergent promoters traverses the antisense TSSs and extends beyond the promoters (Extended Data Fig. 6).

Since transcriptional dynamics may differ at highly transcribed loci with an increased polymerase (Pol) II loading, we separated the convergent promoters into three categories based on the expression level of the host RNA. Previous analyses indicated that higher host RNA expression is associated with reduced daRNA expression^18^, indicating that a potential transcriptional interference is resolved in favor of the host RNA. In contrast, our dynamic expression data show that the correlation of TSSs at higher-expressed host RNAs was not different from that at lower-expressed loci (Fig. 1h and Extended Data Figs. 3d and 4d).

In addition to host gene expression, we reasoned that a greater distance between the two promoters of a convergent promoter pair might affect transcriptional dynamics, as the Pol IIs would spend more time transcribing larger convergent regions. The distance between the convergent TSS2 and TSS3 ranged from 2 to 2,301 bp with a median of 413 bp. Again, convergent transcription between convergent promoters showed a positive correlation regardless of the distance between the two constituent promoters (Fig. 1i and Extended Data Figs. 3e and 4e).

These results indicate that two convergent promoters are more likely to be co-regulated jointly in the same direction than to interfere with each other, challenging the model of transcription interference in numerous instances.

### Cotranscription from convergent promoters

**Fig. 2:**
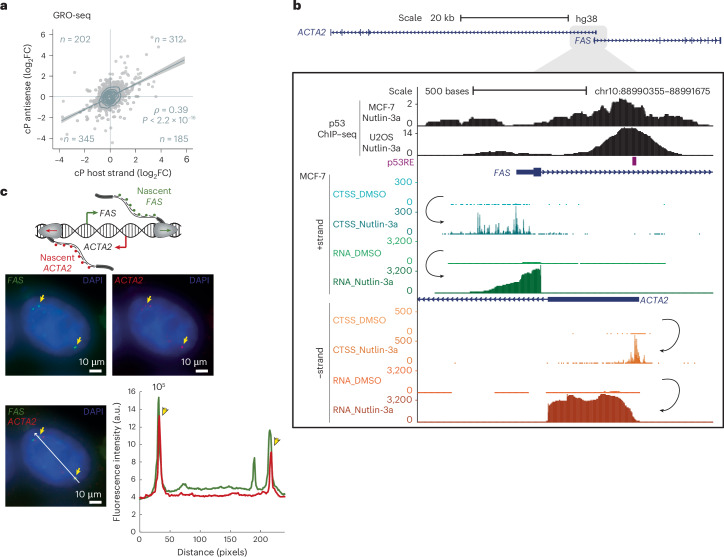
Simultaneous transcription from the convergent *ACTA2* and *FAS* promoters. **a**, Differential GRO-seq data from Nutlin-3a and DMSO control-treated MCF-7 cells (GSE53499) at convergent promoter regions’ sense and antisense strand. Linear regression with 95% confidence intervals. Spearman correlation with two-tailed significance. **b**, UCSC genome browser image of the 5′-overlapping *ACTA2* (−strand) and *FAS* (*+*strand) genes. The top tracks display p53 chromatin immunoprecipitation sequencing (ChIP–seq) data and the p53 response element (p53RE). The bottom tracks display CAGE–seq-detected TSS (CTSS), and RNA-seq counts on the *+*strand (*FAS*) and the *−*strand (*ACTA2*) from MCF-7 cells. Nutlin-3a treatment substantially increased CTSS and RNA-seq counts at both the *+*strand and the −strand. **c**, The first intronic sequences of *FAS* and *ACTA2* have been dual-color labeled using smFISH. Microscopy images display expression of nascent *FAS* (green; left top image), *ACTA2* (red; right top image) and their overlap (lower image) in Nutlin-3a-treated MCF-7 cells. The fluorescent intensity profiles at the region of interest (white line/arrow in lower image) highlight the overlap of nascent *FAS* and *ACTA2* expression, which provides evidence for their cotranscription from the same loci (lower right panel). Extended Data Fig. 5c is an additional example. Approximately 24% of the assessed single cells displayed overlapping FAS and ACTA2 expression signals. DAPI, 4,6-diamidino-2-phenylindol. Source data

CAGE–seq and RNA-seq measure predominantly mature RNA that has undergone several post-transcriptional processes. To corroborate our findings on nascent RNA, we used global run-on sequencing (GRO-seq) data from Nutlin-3a and DMSO control-treated MCF-7 cells. Similar to the CAGE–seq and RNA-seq data (Fig. 1e–g and Extended Data Fig. 5a,b), transcription between convergent promoters also showed a significant positive correlation in GRO-seq analyses, albeit to a lesser extent, probably due to biological and technical variation (Fig. 2a and Extended Data Fig. 5c). However, as CAGE–seq, RNA-seq and GRO-seq data were generated from bulk cells, it remained unclear whether converging polymerases transcribe in opposite directions at the same locus of the same allele and cell. To resolve this, we utilized convergent promoters that initiate the transcription of converging mRNAs, of which we identified 97 (RPE-1) to 142 (U2OS), such as the *FAS*/*ACTA2* locus. The upstream proximal promoters of *FAS* and *ACTA2* constitute a convergent promoter that gives rise to the *FAS* and *ACTA2* mRNAs. *FAS* (*+*strand) is activated through an intronic p53 binding site upstream of the *ACTA2* TSS (−strand)^42^. *FAS* overlaps 5′ with *ACTA2*, which is another p53 target^43^. CAGE–seq and RNA-seq data show upregulation of *FAS* and *ACTA2* upon p53 activation (Fig. 2b). The model of transcription interference stipulates that the two converging mRNAs could not be transcribed from the same locus at the same time because of Pol II collision^23^ or the perturbation of a promoter by one of the traversing Pol IIs^20^. To obtain locus-specific transcription information, we employed single-molecule fluorescent in situ hybridization (smFISH). Using dual-color labeling of the first intronic sequence of *FAS* and *ACTA2*, we detected nascent RNAs at TSSs of both genes upon Nutlin-3a treatment. MCF-7 is a polyploid cell line, so we observed multiple active TSSs per cell. Intriguingly, we identified cotranscription of *FAS* and *ACTA2* from the same locus, that is, overlapping signals, occurring in 24% of single cells (Fig. 2c and Extended Data Fig. 5d). These data provide evidence that convergent transcription can occur at the same locus without diminishing promoter productivity. In defiance of the transcription interference model, these data provide further evidence that convergent promoters are co-regulated in the same direction and that p53 binding to the *ACTA2* promoter facilitates activation of the neighboring *FAS* promoter.

### Transcription factors co-regulate convergent promoters

**Fig. 3:**
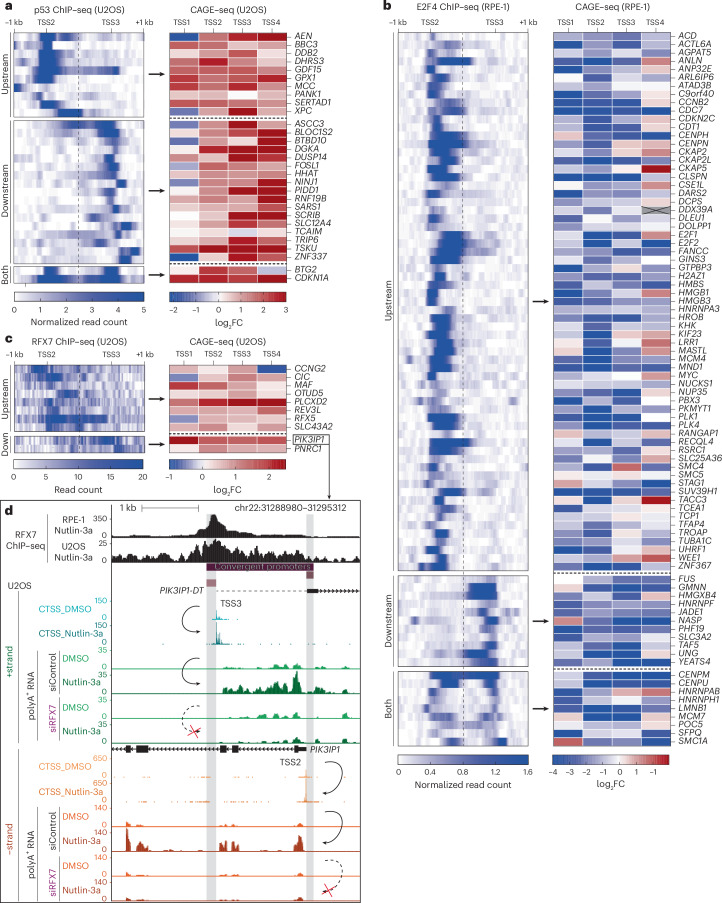
Transcription factors co-regulate convergent promoters. **a**–**c**, Heatmaps of transcription factor binding signals (left panels) and log_2_FC (Nutlin-3a versus DMSO control) at CAGE–seq peaks harboring TSSs (right panels) displayed for convergent promoters bound by p53 (**a**), E2F4 (**b**) and RFX7 (**c**). The convergent promoters are sorted by the occurrence of transcription factor peaks near the upstream (TSS2) or downstream (TSS3) promoter. **d**, UCSC genome browser image of the 5′-overlapping *PIK3IP1* (−strand) and *PIK3IP1-DT* (*+*strand) genes elongated to the correct TSS (dotted line). *PIK3IP1* is *trans*-activated by the p53-induced transcription factor RFX7 (ref. ^45^). The top tracks display RFX7 ChIP–seq data^45^ and the identified convergent promoters. The bottom tracks display CAGE–seq-detected TSS (CTSS) and RNA-seq counts on the *+*strand (*PIK3IP1-DT*) and the −strand (*PIK3IP1*) from U2OS cells. The location of TSS1 is displayed by the right edge of the gray highlighted *PIK3IP1* subpromoter, while TSS4 is shown at the left edge of the gray highlighted *PIK3IP1-DT* subpromoter. Nutlin-3a treatment substantially increased CTSS and RNA-seq counts at both the *+*strand and the −strand. Depletion of RFX7 by siRNA abrogated the Nutlin-3a-mediated increase in RNA-seq signal at both strands. Source data

**Extended Data Fig. 7:**
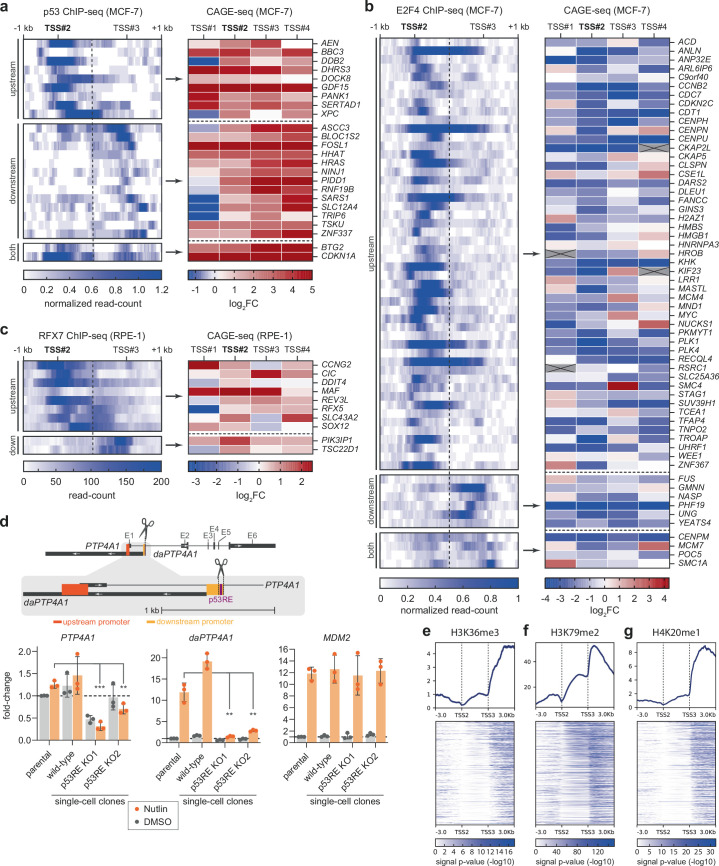
Transcription factors co-regulate convergent promoters. **(a-c)** Heatmaps of transcription factor binding signals (left panels) and log2FC (Nutlin-3a vs DMSO control) at CAGE-seq peaks harboring TSSs (right panels) displayed for convergent promoters bound by **(a)** p53, **(b)** E2F4, and **(c)** RFX7. The convergent promoters are sorted by the occurrence of transcription factor peaks near the upstream (TSS#2) or downstream promoter (TSS#3). Complementary to Fig. 3a-c. **(d)** The *PTP4A1* gene locus harbors *PTP4A1* on the *+strand* and its downstream antisense RNA (*daPTP4A1*) on the *-strand* (upper panel). CRISPR/Cas9 has cut the p53RE located in the downstream promoter in RPE-1 cells. RT-qPCR data from parental RPE-1 cells, a wild-type clone, and two homozygous p53RE knock-out (KO) clones treated with Nutlin-3a or DMSO control (bottom panels). Expression has been normalized to *GAPDH* and DMSO control-treated parental cells. *MDM2* expression served as positive control. Mean and standard deviation are displayed. Statistical significance was obtained through a two-sided t-test; n = 3 biological replicates. **(e-g)** Summary profiles (top panels) and heatmaps with individual convergent promoter regions (bottom panels) display epigenetic signals at MCF-7 convergent promoters. Convergent promoters are length-sorted in descending order. **(e)** H3K36me3, **(f)** H3K79me2, and **(g)** H4K20me1 signal p-values (-log10) at scale-adjusted convergent promoter regions. Negative decadic logarithms of signal p-values computed using a Poisson model-based statistical test were directly obtained from ENCODE data files. Source data

Given that p53 binding to the proximal *ACTA2* promoter is associated with activation of the neighboring *FAS* promoter (Fig. 2a), we asked to what extent convergent co-regulated promoters may, in fact, broaden the width and spectrum of regulatory sequence around the start site of a gene. To investigate this situation, we used a list of 343 p53 targets^43^ and selected those with a convergent promoter structure bound by p53. Intriguingly, we observed that p53-bound convergent promoters displayed predominantly an upregulation of both promoter parts regardless of whether p53 engaged with the upstream or downstream promoter (Fig. 3a and Extended Data Fig. 7a).

We wondered whether this was an ability unique to p53 or a more common feature of transcription factors. Notably, the p53 gene regulatory network contains multiple transcription factors^44^. While p53 typically upregulates the genes it binds to, p53-mediated downregulation occurs indirectly, for example, through the cell cycle *trans*-repressor complex DREAM (DP, RB-like, E2F4 and MuvB)^38^. Therefore, we used a list of DREAM target genes^38^ and selected those with convergent promoters bound by the key DREAM component E2F4. In line with our results for p53, we found that E2F4/DREAM-bound convergent promoters showed primarily a downregulation of both constituent promoters regardless of whether E2F4/DREAM bound to the upstream or downstream promoter (Fig. 3b and Extended Data Fig. 7b).

In addition to p53 and E2F4, we investigated convergent promoters bound by RFX7, a p53-induced transcription factor^45^. Again, RFX7-bound convergent promoters displayed an upregulation of both promoters regardless of which one of them was bound by RFX7 (Fig. 3c and Extended Data Fig. 7c). For instance, the RFX7 target *PIK3IP1* (−strand) is indirectly activated by p53 through RFX7 (ref. ^45^) and a convergent promoter initiates the transcription of *PIK3IP1-DT* on the *+*strand. Specifically, RFX7 binds to the proximal promoter of *PIK3IP1-DT* downstream of the *PIK3IP1* TSS. Depletion of RFX7 by siRNA abrogated the Nutlin-3a-mediated upregulation of both *PIK3IP1* and *PIK3IP1-DT* (Fig. 3d). This finding underscores that convergent promoter structures enable transcription factors bound to antisense promoters located hundreds of base pairs downstream to affect transcription from the proximal upstream promoter and vice versa.

**Fig. 4:**
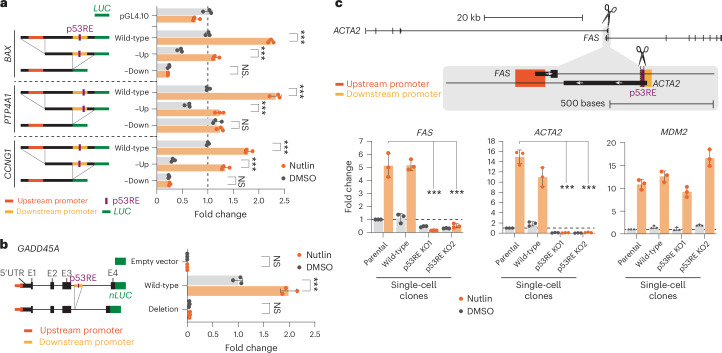
Convergent promoters co-regulate each other. **a**, Dual-luciferase assay in Nutlin-3a and DMSO control-treated U2OS cells. Convergent promoter regions of the p53 target genes BAX, PTP4A1 and CCNG1 drove firefly luciferase expression. Upstream and downstream promoters have been removed in the respective constructs. **b**, *GADD45A* gene fused with nanoluciferase. Dual-nanoluciferase assay in Nutlin-3a and DMSO control-treated U2OS cells. The downstream promoter has been removed in the respective construct. Data in **a** and **b** are normalized to wild-type activity in DMSO-treated cells. **c**, The *FAS/ACTA2* gene locus harbors *FAS* on the *+*strand and *ACTA2* on the −strand (upper panel). CRISPR–Cas9 has cut the p53RE located in the ACTA2 promoter in U2OS cells. RT-qPCR data from parental U2OS cells, a wild-type clone and two homozygous p53RE knock-out (KO) clones treated with Nutlin-3a or DMSO control (bottom panels). Expression has been normalized to *GAPDH* and DMSO control-treated parental cells. *MDM2* expression served as positive control. Data in **a**–**c** are shown as mean ± s.d. Statistical significance was obtained through a two-sided *t*-test; *n* = 3 biological replicates. *P < 0.05, **P < 0.01, ***P < 0.001; NS, nonsignificant. Source data

To validate the existence of a regulatory interdependence of convergent promoters, we took a three-pronged approach. First, we cloned three genomic regions containing convergent promoters controlling the expression of the p53 target genes *BAX*, *PTP4A1* and *CCNG1* and evaluated their activity in luciferase reporter gene assays. All three regions showed p53 binding to the downstream antisense promoter and conferred increased reporter gene expression upon Nutlin-3a treatment. Interestingly, removing the p53RE-containing downstream promoter driving TSS3 abolished the increased expression upon Nutlin-3a treatment. In contrast, removing the upstream promoter associated with TSS2 reduced the overall activity only of the convergent promoter regions (Fig. 4a). These results indicate that the downstream promoter mediates the p53 response in each of the three regions and positively affects its upstream counterparts. Second, given that none of the three cloned regions contained the entire sequence of their respective genes, we tested the regulation of a whole gene. We identified a convergent promoter constellation in the p53 target *GADD45A*, which is only about 3.1 kb in size and harbors the downstream promoter in the third intron of the gene. Therefore, we used a luciferase reporter system containing the *GADD45A* gene locus with a translationally fused nanoluciferase. In strong support of our hypothesis, the *GADD45A* gene reporter was activated upon Nutlin-3a treatment, and this activation was abolished when the downstream promoter was deleted. In fact, deleting the downstream promoter in the third intron abolished most of the reporter activity, underscoring the critical role of downstream promoters for the host gene regulation—even if located in introns (Fig. 4b). Although the *GADD45A* minigene reporter provided gene context, it still lacked the native chromatin context. To address this, we third used CRISPR–Cas9 to mutate the p53RE in the downstream antisense promoter located in the first intron of *FAS* and *PTP4A1*. Consistent with the results obtained with the reporter gene systems, homozygous mutation of the p53RE led to a reduction in the expression of both the host genes *FAS* and *PTP4A1* and the downstream antisense transcripts *ACTA2* and *daPTP4A1* that are induced from the downstream promoters (Fig. 4c and Extended Data Fig. 7d).

Collectively, convergent promoters seem to provide a molecular architecture enabling transcription factors to co-regulate juxtaposed promoters.

### An active chromatin signature marks convergent promoters

**Fig. 5:**
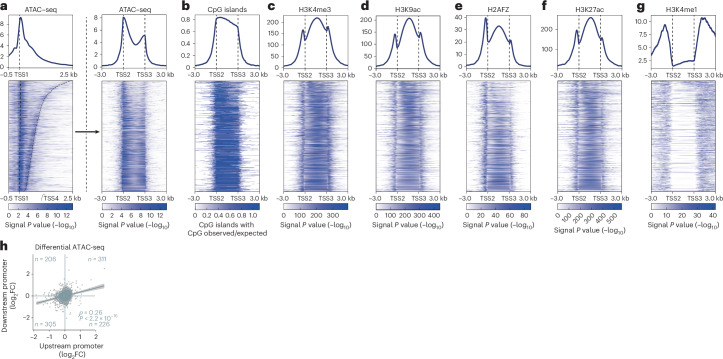
An active chromatin structure connects convergent promoters. **a**–**g**, Summary profiles (top panels) and heatmaps with individual convergent promoter regions (bottom panels) display epigenetic signals at MCF-7 convergent promoters. Convergent promoters are length-sorted in descending order. **a**, ATAC–seq signals. Left, original scale with start (TSS1) and end (TSS4) indicated by dashed lines. Right, all regions adjusted to the same scale with start (TSS2) and end (TSS3) marked by dashed lines. **b**–**g**, CpG islands (**b**), H3K4me3 (**c**), H3K9ac (**d**), H2AFZ (**e**), H3K27ac (**f**) and H3K4me1 (**g**) signal *P* values (−log_10_) at scale-adjusted regions. Negative decadic logarithms of signal *P* values computed using a Poisson model-based statistical test were obtained directly from ENCODE data files. **h**, Differential ATAC–seq signal (log_2_FC Nutlin-3a compared with DMSO control) at downstream promoter compared with its upstream promoter counterpart in MCF-7 cells. Linear regression with 95% confidence intervals. Spearman correlation with two-tailed significance. Source data

To better understand the architecture of convergent promoters, we examined the chromatin structure at the respective loci and the surrounding area using data from ENCODE. Assay for transposase-accessible chromatin with sequencing (ATAC–seq) data indicate open chromatin, that is, nucleosome-depleted regions, at both constituent promoters. In agreement with earlier observations^17–19^, a reduced ATAC–seq signal in between the promoters suggests the presence of nucleosomes separating two nucleosome-free promoter regions (Fig. 5a). Interestingly, convergent promoters largely overlap CpG islands (Fig. 5b), consistent with the high GC content previously observed between convergent promoters^17,18^. Moreover, the active marks H3K4me3, H3K9ac and H3K27ac are enriched at the promoter regions proximal to the TSSs and display a maximum at the nucleosomes between the convergent promoters. Similarly, we observe a similar distribution of H2AFZ (Fig. 5c–f). Notably, the promoter marks H3K4me3, H3K9ac, and H2AFZ^46^ between TSS2 and TSS3 distinguish these promoter regions from enhancers. While H3K27ac is found frequently at promoters and enhancers, H3K4me1 is an enhancer mark typically not found at promoters but only at promoter flanking regions^46^. The regions spanning convergent promoter constellations are devoid of the enhancer mark H3K4me1. Still, their flanking regions are enriched for H3K4me1 (Fig. 5g). The transcription-elongation marks H3K36me3, H3K79me2 and H4K20me1 are enriched towards the host gene body (Extended Data Fig. 7e–g), consistent with their known enrichment at coding sequences^46^.

**Extended Data Fig. 8:**
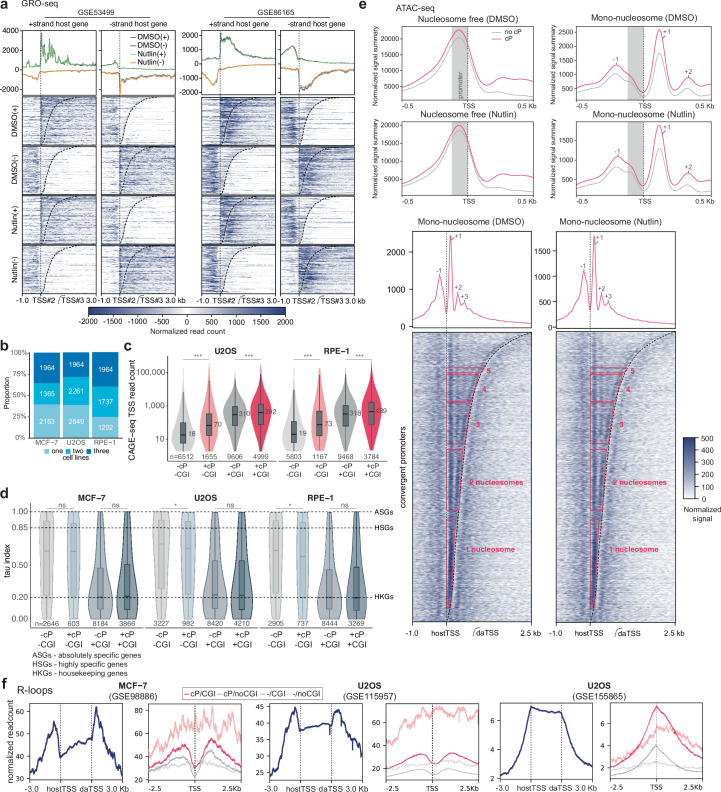
Transcriptional characteristics at convergent promoters. **(a)** Summary profiles (top panels) and heatmaps with individual convergent promoter regions (bottom panels) display GRO-seq signals at MCF-7 convergent promoters separated by host gene strand. GRO-seq data of Nutlin-3a and DMSO control-treated MCF-7 cells obtained from GSE86165 and GSE53499. **(b)** Convergent promoters were identified using only two convergent CAGE-seq peaks overlapping a GENCODE-annotated TSS in the respective cell lines, and the proportion was identified in one, two, or all three cell lines. **(c)** Violin plots summarize the read-count values at GENCODE-annotated TSS-overlapping CAGE-seq peaks that are part of convergent super-promoters ( + cP; red) or not (-cP; grey) and that overlap with CGIs ( + CGI, dark) or not (-CGI, light) in U2OS and RPE-1 cells. Statistical significance was assessed using a two-sided, unpaired Wilcoxon rank sum test. p-value *** < 0.001. **(d)** Violin plots summarize the TAU index of GENCODE-annotated TSS-overlapping CAGE-seq peaks (derived from MCF-7, U2OS, or RPE-1) that are part of convergent super-promoters ( + cP) or not (-cP) and that overlap with CGIs ( + CGI, dark) or not (-CGI, light). Statistical significance was assessed using a two-sided, unpaired Wilcoxon rank sum test. p-value * < 0.05. **(c,d)** Boxes show the median, upper, and lower quartiles, whiskers 1.5x interquartile range. **(e)** ATAC-seq data from Nutlin-3a and DMSO control-treated MCF-7 cells separated into nucleosome-free (fragment size <100 bp) and mono-nucleosome reads (fragment size 180-240 bp) at GENCODE-annotated TSS-overlapping CAGE-seq peaks that are part of convergent super-promoters (cP) or not (no cP). Heatmaps with individual length-sorted convergent promoter regions (bottom panels) display mono-nucleosome signals at MCF-7 convergent promoters **(f)** Summary profiles of read-counts from MCF-7 and U2OS DRIP-seq data for the extended set of scale-adjusted convergent promoters (left panels) and at TSSs from CAGE-seq peaks that overlap GENCODE-annotated TSSs and that are part of convergent promoters (cP; red) or not (-; grey) and that overlap with CGIs ( + CGI, dark) or not (-CGI, light) (right panels). Source data

Since current data suggest that two converging Pol II cannot bypass each other^23^, we assessed Pol II pausing at convergent promoters. Indeed, GRO-seq signals indicate increased Pol II pausing starting at TSS2 and tending to extend all the way to TSS4 (Extended Data Fig. 8a). Thus, our data show a comparably long region with Pol II pausing in the sense direction. In the antisense direction, there was increased pausing starting at TSS1 but rather little between TSS3 and TSS1, suggesting that Pol II pausing at TSS3 did not occur frequently. Consequently, Pol II residence time on the antisense strand appears lower.

To test whether the co-regulation within convergent promoters extends to common changes in the chromatin structure, we generated and analyzed differential ATAC–seq data. In support of a joint regulation, ATAC–seq signals at both constituent promoters of the convergent promoter structure displayed a significant positive correlation in response to Nutlin-3a treatment (Fig. 5h).

### Characteristics of convergent promoters

**Fig. 6:**
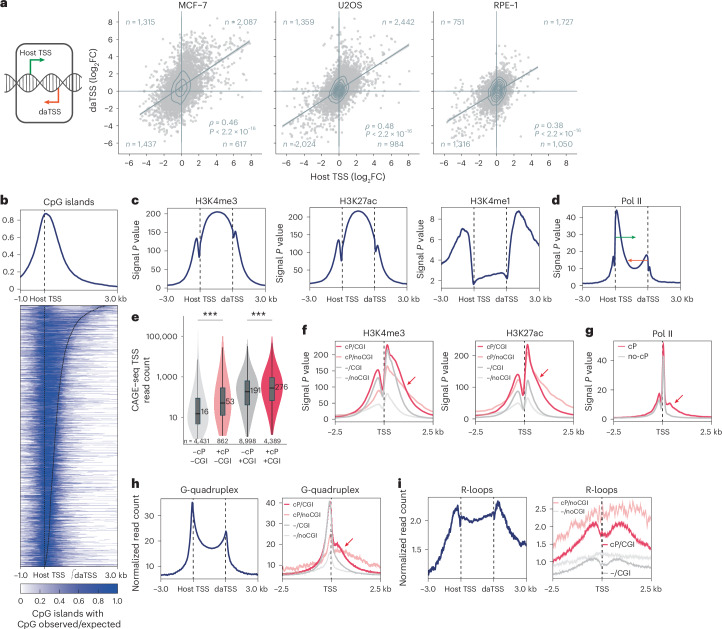
Characteristics of convergent promoters. **a**, Schematics (left) highlight the TSSs that have been used to identify the extended set of convergent promoters and that have been compared for their log_2_FC (Nutlin-3a compared with DMSO control). Spearman correlation in MCF-7 (left panel), U2OS (middle panel) and RPE-1 cells (right panel). Linear regression with 95% confidence intervals. Spearman correlation with two-tailed significance. **b**, Summary profile (top panel) and heatmap with individual convergent promoter regions (bottom panels) starting from the host TSS display CpG island signals. The extended set of MCF-7 convergent promoters is length-sorted in descending order. **c**,**d**,**f**,**g**, ENCODE data from MCF-7 cells^57^. Summary profiles of H3K4me3 (left panels **c** and **f**), H3K27ac (middle panel **c**, right panel **f**), H3K4me1 (right panel **c**) and Pol II (**d**,**g**) signals for the extended set of scale-adjusted MCF-7 convergent promoters. **e**, Violin plots summarize the read counts at GENCODE-annotated TSS-overlapping CAGE–seq peaks that are part of convergent promoters (+cP, red) or not (−cP, gray) and that overlap with CGIs (+CGI, dark) or not (−CGI, light) in MCF-7 cells. Statistical significance was assessed using a two-sided, unpaired Wilcoxon rank sum test. ***P < 0.001. Boxes show the median, upper and lower quartiles, whiskers 1.5x interquartile range. **f**,**g**, Summary profiles of H3K4me3 (left panel **f**), H3K27ac (right panel **f**) and Pol II (**g**) at TSSs from CAGE–seq peaks that overlap GENCODE-annotated TSSs and that are part of convergent promoters (cP, red) or not (no-cP, gray) and that overlap with CGIs (CGI, dark) or not (noCGI, light). **h**,**i**, Summary profiles of read counts from U2OS G4 ChIP–seq (GSE162299) (**h**) and MCF-7 DRIP–seq (GSE81851) (**i**) data for the extended set of scale-adjusted convergent promoters (left panels) and at TSSs from CAGE–seq peaks that overlap GENCODE-annotated TSSs and that are part of convergent promoters (cP, red) or not (−, gray) (right panels) and that overlap with CGIs (+CGI, dark) or not (−CGI, light). Source data

**Extended Data Fig. 9:**
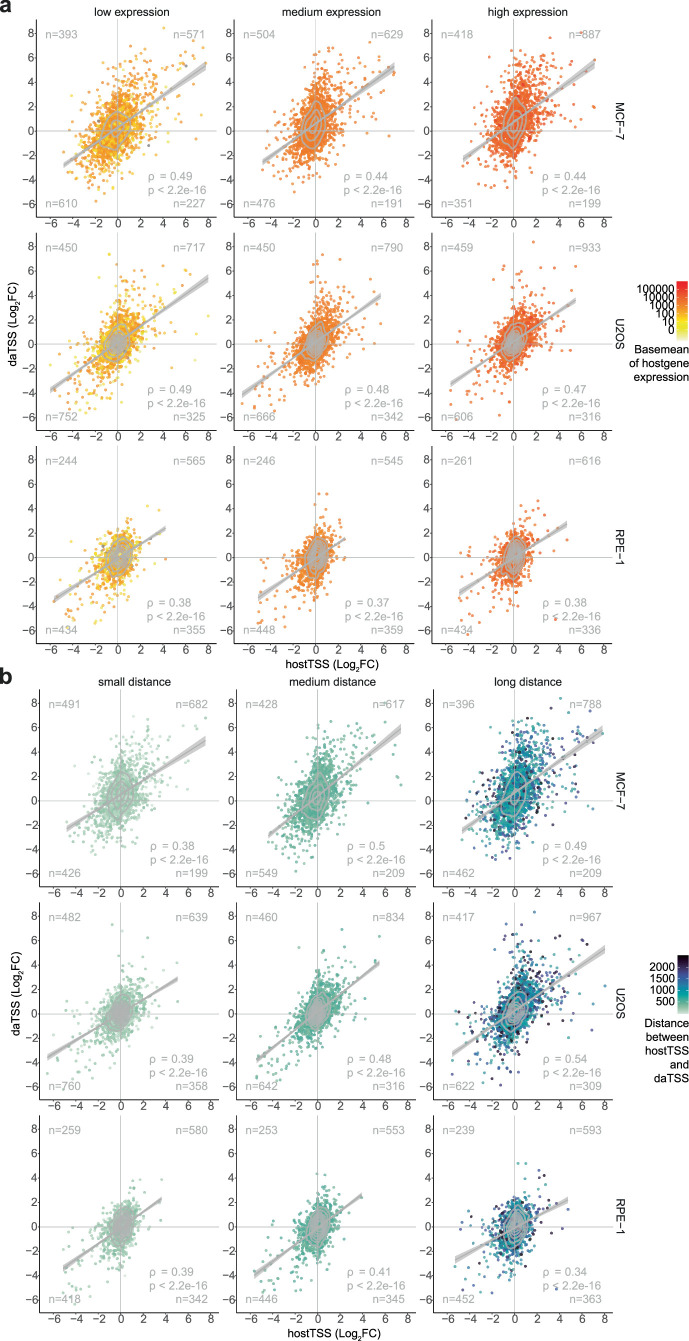
Positive correlation independent of base expression and TSS distance. The extended set of convergent promoters has been separated into three groups based on **(a)** the basemean expression of host gene expression (elicited by hostTSS) and **(b)** the distance between hostTSS and daTSS. All three groups display a similar positive Spearman correlation of the expression dynamics. Data from MCF-7 (upper panels), U2OS (middle panels), and RPE-1 cells (bottom panels). Linear regression with 95% confidence intervals. Spearman correlation with two-tailed significance. Source data

Previous studies have suggested that the promoters of about a quarter of expressed genes are affected by convergent transcription. To identify such genes, we searched for pairs of host gene TSSs and daTSSs (corresponding to TSS2 and TSS3 above)^17–19,26^—a strategy significantly less conservative than the one pursued here, that is, searching for pairs of divergent TSS pairs (Fig. 1d and Extended Data Fig. 1a). To optimize the sensitivity of our approach, we directly paired convergent CAGE peaks. We then selected all pairs overlapping with a TSS of a GENCODE-annotated gene. The dominant TSS was defined as the host TSS (equivalent to TSS2 above). This more sensitive search identified an extended set of ~4,800 to ~6,800 convergent promoter constellations (Supplementary Tables 5–8). We were able to identify many of them in several cell systems (Extended Data Fig. 8b). The TSSs in the extended set of convergent promoters also displayed a positive correlation of expression (Fig. 6a). Again, the positive correlation of convergent transcription was not affected by host gene expression levels (Extended Data Fig. 9a) or the distance between the TSSs (Extended Data Fig. 9b). Moreover, the extended set of convergent promoters also exhibits a typical chromatin structure spanning the juxtaposed TSSs and including CpG islands (CGIs; Fig. 6b). We found enrichment for H3K4me3 and H3K27ac and depletion for H3K4me1 (Fig. 6c). Pol II occupancy data corroborated Pol II loading in a converging direction (Fig. 6d).

To directly compare the characteristics of convergent promoter TSSs with other TSSs, we filtered for all CAGE–seq peaks overlapping with a GENCODE-annotated TSS. We found that 24.5–29.2% of CAGE–seq peaks supported by GENCODE TSSs were part of convergent promoters. Given that convergent promoters enrich for CGIs (Fig. 4j) and CGI promoters represent an important promoter class^47,48^, we separated them into CGI-overlapping and non-CGI TSSs. Convergent promoter TSSs showed a significantly higher expression than regular promoter TSSs (Fig. 6e and Extended Data Fig. 8c), contrasting earlier studies that associated convergent transcription at promoters with lower-expressed genes^17^. By contrast, tissue specificity based on FANTOM5 data^40^ did not differ between convergent promoter TSSs and regular promoter TSSs (Extended Data Fig. 8d). Likewise, our ATAC–seq data indicate that nucleosome positioning near TSSs of convergent promoters is similar to other promoter TSSs (Extended Data Fig. 8e). However, in agreement with their higher productivity, the convergent promoter TSSs displayed more pronounced signals for the activity-associated marks H3K4me3 and H3K27ac (Fig. 6f), while Pol II occupancy was similar to other promoter TSSs (Fig. 6g). A characteristic feature of convergent promoter TSSs is a broader signal for H3K4me3, H3K27ac and Pol II occupancy, extending particularly downstream (Fig. 6f,g). In fact, we found that this broader signal is indicative of the adjacent downstream TSSs. Further, we discovered that G-quadruplex (G4) structures, which can form at nucleosome-depleted and GC-rich DNA^49^, show a broader signal at convergent promoter TSSs when compared with other TSSs (Fig. 6h). Given that antisense transcription has been associated with R-loop formation^50^, for example, at the promoters of *VIM*^34^ and *TCF21* (ref. ^36^), we assessed the prevalence of R-loops at convergent promoters. We found that convergent promoter TSSs were enriched for R-loop formation compared with other promoter TSSs (Fig. 6i and Extended Data Fig. 8f), indicating an association between convergent promoters and R-loops that may be of functional importance.

Collectively, our data indicate that convergent promoters may regulate more than a quarter of all active gene TSSs. We find that convergent promoters are characterized by strong and broad active promoter marks and G4 structures and are enriched for R-loops. Critically, convergent promoter TSSs are significantly more productive.

### Annotation of daRNAs initiated from 2,158 host genes

**Fig. 7:**
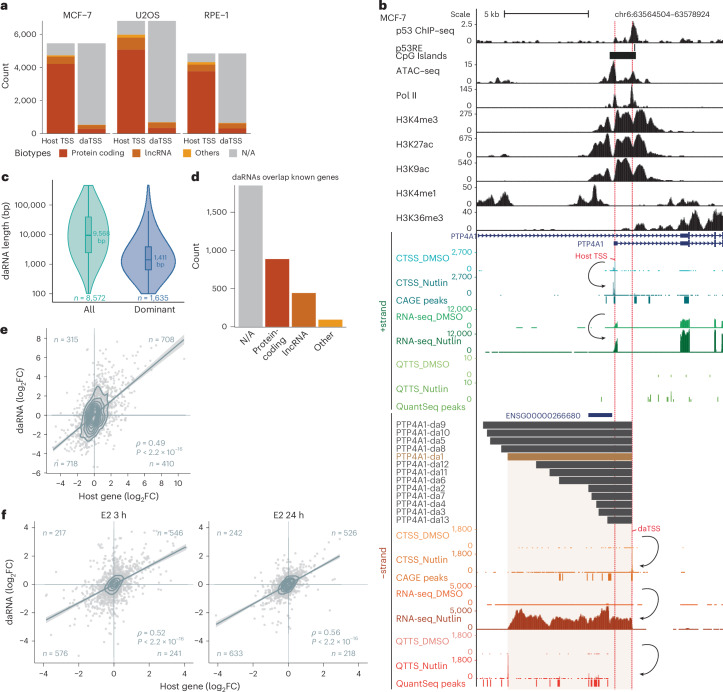
Downstream antisense RNAs initiated from 1,635 host genes are co-regulated with their host genes through convergent promoters. **a**, Biotypes associated with GENCODE-annotated TSSs that overlap with the CAGE–seq peaks harboring the respective convergent promoter TSSs. The vast majority of daTSS peaks overlapped no GENCODE-annotated TSS. N/A, not available. **b**, UCSC genome browser image of the *PTP4A1* gene (+strand) and its 5′-overlapping antisense RNAs (daRNAs; −strand). *PTP4A1* is *trans*-activated by p53. The top tracks display p53 ChIP–seq data and the p53 response element (p53RE)^58^ as well as ATAC–seq, Pol II, H3K4me3, H3K27ac, H3K9ac, H3K4me1 and H3K36me3 data from ENCODE^57^. The bottom tracks display CAGE–seq-detected TSS (CTSS), RNA-seq and QTTS counts on the *+*strand (*PTP4A1*) and the −strand (*PTP4A1_daRNAs*) from MCF-7 cells. Nutlin-3a treatment substantially increased CTSS, RNA-seq and QuantSeq counts at both the *+*strand and the −strand. Downstream antisense RNAs (daRNAs) initiated from the convergent promoter at *PTP4A1* are indicated in gray. The daRNAs are numbered according to the order of the associated QTTS count, starting from the highest count (*PTP4A1-da1*; dominant daRNA; golden colored). **c**, Violin plots summarize the length of all daRNAs and the dominant daRNAs initiated from the extended set of convergent promoters in MCF-7 cells. Boxes show the median, upper and lower quartiles, whiskers 1.5x interquartile range. **d**, Biotypes of annotated genes that are included (overlap) in the daRNAs or into which daRNAs read-in (merge with). **e**,**f**, RNA-seq-derived differential expression of dominant daRNAs (*y* axis) compared with the differential expression of their respective host genes (*x* axis) in 24 h Nutlin-3a-treated (**e**) and 3 h (GSE117942) and 24 h (GSE173976) estradiol (E2)-treated MCF-7 cells (**f**). Linear regression with 95% confidence intervals. Spearman correlation with two-tailed significance. Source data

**Extended Data Fig. 10:**
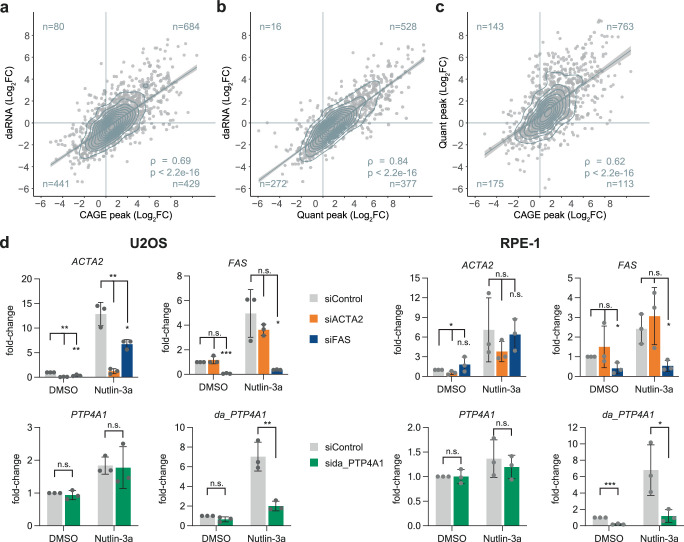
Validation of differential daRNA expression. Differential expression measured by RNA-seq (y-axis) displays a positive Spearman correlation with **(a)** differential expression measured by CAGE-seq (x-axis) and **(b)** differential expression measured by QuantSeq (x-axis). **c,** Differential expression measured by QuantSeq (y-axis) displays a positive Spearman correlation with expression measured by CAGE-seq (x-axis) for the identified daRNAs. Differential expression measured by RNA-seq (y-axis) displays a positive Spearman correlation for the identified daRNAs. Data from MCF-7. Linear regression with 95% confidence intervals. Spearman correlation with two-tailed significance. **(d)** RT-qPCR data of *ACTA2*, *FAS*, *da_PTP4A1*, and *PTP4A1* from U2OS and RPE-1 cells transfected with indicated siRNAs and treated with Nutlin-3a or DMSO solvent control, normalized to siControl DMSO treatment and *ACTR10* expression. Mean and standard deviation are displayed. Statistical significance was obtained through a two-sided t-test; n = 3 biological replicates. p-value ** < 0.01 and *** < 0.001. Source data

The daRNAs represent a subclass of NATs, namely 5′-overlapping *cis*-NATs, which were previously believed to be rare^12^. Similar to *PIK3IP1-DT*, for which the actual TSS is not part of the current gene annotation (Fig. 3d), we found that the overwhelming majority of daTSSs did not overlap any GENCODE-annotated TSS (Fig. 7a). Thus, daRNAs appear to be largely missing from the annotation. To annotate daRNAs, we complemented CAGE–seq and RNA-seq with 3′ RNA-seq, that is, QuantSeq data^51^, to determine transcription termination sites (TTSs). Based on these three data layers, we identified daRNAs initiated from 2,158 host genes, 1,635 which were missing in GENCODE. Annotation of the primary daRNA transcript involves the detection of its most pronounced QuantSeq signal (Supplementary Table 9; Methods). For instance, *PTP4A1* is regulated by a convergent promoter generating daRNAs with different transcript lengths (Fig. 7b). daRNAs had a median length of 9,568 bp (Fig. 7c), and 97% of all daRNAs extended across the host TSS without any apparent Pol II blockade. Strikingly, daRNAs overlapped many annotated lncRNAs and protein-coding genes (Fig. 7d). Thus, our data indicate an incomplete annotation and the existence of alternative start sites, for instance, in the case of *PIK3IP1-DT* (Fig. 3d). We combined the annotation with our RNA-seq data to assess differential expression changes of the host genes and their respective daRNAs. Differential RNA-seq analysis corroborated the positive correlation of 5′-overlapping sense and antisense transcripts (Fig. 7d). Notably, QuantSeq, CAGE–seq and RNA-seq data displayed a positive correlation (Extended Data Fig. 10a–c).

Since antisense RNAs affect adjacent genes^14,52,53^, we examined whether the expression of *FAS*/*ACTA2* or *PTP4A1*/*da_PTP4A1* affects each other. To this end, we performed a knockdown of the respective RNAs and tested the expression of their mates. Although we observed substantial knockdown of the targeted RNAs, we did not observe a consistent effect on the 5′-overlapping RNA counterpart (Extended Data Fig. 10d). These data are in agreement with other studies that have not found a universal contribution of overlapping antisense transcripts to each other’s expression^14^ and that have found *cis*-regulatory elements to be of potentially greater relevance in controlling nearby genes^54^.

With the daRNA annotations at hand, we could test whether convergent promoters elicit co-regulation of convergent transcription also under conditions that do not involve activation of p53. We utilized data from estradiol (E2)-treated MCF-7 cells. We additionally observed a positive correlation of daRNA and host RNA expression in response to 3 h and 24 h E2 treatment (Fig. 7f), suggesting a more universal co-regulation of 5′-overlapping transcription through convergent promoters.

## Discussion

Our work reveals an unexpected co-regulation of convergent promoters, that is, a joint regulation in the same direction. The convergence of sense and antisense transcription was believed to cause transcription interference^22,24^, either because two converging Pol II complexes collide and cannot bypass each other^23^ or because Pol II passage would perturb one of the promoters^20^. Instead, we found that convergent transcription marks juxtaposed promoters that can be co-regulated in the same direction. In fact, the expression correlation between convergent TSSs was essentially as strong as that between divergent TSSs, for which co-regulation is commonly observed and widely accepted (Fig. 5i and Extended Data Fig. 1c). Whereas the convergent promoters are located in distinct nucleosome-depleted regions that are separated by nucleosomes (Extended Data Fig. 8e), they appear to be linked epigenetically by CpG islands (CGIs) and active promoter marks that show peak signal between the constituent promoters (Fig. 5a–f and j–k).

Most importantly, we find that transcription factor binding to any individual subpromoter can be sufficient to co-regulate all TSSs in this structure. Thus, convergent co-regulated promoters (cocoProms) substantially expand our notion of the promoter architecture, with important ramifications for our understanding of gene regulation. For decades, researchers have focused on proximal promoters and adjacent upstream regions to identify binding sites of transcription factors and other features potentially affecting gene regulation. However, the co-regulation of convergent promoters, such as *FAS* and *GADD45A* expression regulated by p53 binding to the downstream antisense promoter (Fig. 4) and *PIK3IP1* expression regulated by RFX7 binding to the downstream antisense promoter (Fig. 3d), suggests that gene expression can be affected by promoters several hundred base pairs downstream of a TSS. This finding is thus prompting us to rethink the strategies we use to identify target genes of transcription factors. Notably, the co-regulation is not limited to each constituent promoter acting as an enhancer for the other. Our data show that transcriptional repressors such as E2F4 can also downregulate entire cocoProms (Fig. 3b). E2F4 is a crucial component of the multisubunit repressor complex DREAM, which has been proposed to block transcription from promoters by stabilizing +1 nucleosomes^55^. Since the nucleosomes located between convergent promoters are an obstacle to Pol II passage in both sense and antisense, it makes sense that their stabilization would also downregulate transcription from both convergent promoters.

Although downstream antisense promoters share many characteristics with intragenic enhancers, our data provide evidence that they may be best characterized as promoters (Supplementary Discussion). Similarly, convergent promoters can be distinguished from other well-established promoter classes with which they share key characteristics (Supplementary Discussion).

We found that the intragenic promoters are evolutionarily conserved (Extended Data Fig. 2d). From an evolutionary point of view, the four TSSs of prototypical cocoProms (Fig. 1d) grant the flexibility to produce distinct genes and isoforms from one single regulatory locus. The two constituent promoters offer two spatially separated but functionally linked platforms for transcription factor binding and expression control. Recently, it has been suggested that the initiation of divergent transcription is the ground state of new promoters in yeast, and one of the TSSs may be silenced only later^56^. Thus, an intriguing question is whether cocoProms with four or more TSSs are the actual ground state of newly evolved promoters in metazoans with individual TSSs silenced later.

## Methods

The experiments mentioned below did not require approval from a specific ethics board.

### Cell culture, drug treatment and transfection

MCF-7 (ATCC, cat. no. HTB-22) and U2OS (DSMZ, cat. no. ACC 785) cells were grown in high glucose Dulbecco’s modified Eagle’s media (DMEM) with pyruvate (ThermoFisher Scientific). RPE-1 hTERT cells (ATCC, cat. no. CRL-4000) were cultured in DMEM:F12 media (ThermoFisher Scientific). Culture media were supplemented with 10% fetal bovine serum (FBS; ThermoFisher Scientific) and penicillin/streptomycin (ThermoFisher Scientific). In addition, DMEM was supplemented with nonessential amino acids (ThermoFisher Scientific) for culturing MCF-7 cells. Cell lines were tested twice a year for *Mycoplasma* contamination using the LookOut Detection Kit (Sigma Aldrich), and all tests were negative. Cell authentication was performed using morphological validation.

Cells were treated with DMSO solvent control (0.15%; Carl Roth) or Nutlin-3a (10 µM; Sigma Aldrich) for 24 h.

For knockdown experiments, cells were seeded in six-well plates and reverse transfected with 10 nM Silencer Select siRNAs (ThermoFisher Scientific) using RNAiMAX (ThermoFisher Scientific) and Opti-MEM (ThermoFisher Scientific) following the manufacturerʼ protocol. The following silencer select siRNAs (ThermoFisher Scientific) were used: siControl (cat. no. 4390844), *FAS* (cat. no. s1508), *ACTA2* (cat. no. s945), *da_PTP4A1* (forward CCACGUUUCUCAUAAUUAAtt; reverse UUAAUUAUGAGAAACGUGGtt).

### Genome editing

To target the p53 responsive element in the *PTP4A1* downstream promoter, two gRNAs were selected using the CRISPR/Cas9 guide RNA design checker available on Integrated DNA Technologies (IDT) website. Each gRNA oligo (IDT) was annealed with tracer RNAs (tracRNAs) coupled to ATTO 550 or ATTO 647 fluorophores (IDT), allowing for differentiation between the two gRNAs. Following the manufacturer’s protocol, the annealed gRNA complexes were loaded into the recombinant Cas9 enzyme with enhanced specificity (IDT).

The day after cell seeding, RPE-1 cells were transfected with gRNA–Cas9 ribonucleoprotein complexes using Lipofectamine RNAiMAX (ThermoFisher Scientific) according to the manufacturer’s instructions. Two days after transfection, cells with double positive signals for ATTO 550 or ATTO 647 in the top 20% of the population were sorted individually into single wells of 96-well plates. These cells were then cultured to confluence in a 1:1 mixture of fresh and conditioned medium.

The p53 response element in the *ACTA2* promoter has been deleted from U2OS cells by Cytosurge as described previously^59^. In brief, single U2OS cells were seeded and, on the next day, gRNA–Cas9 RNP complexes (20 ng µl^−1^) targeting the *ACTA2* p53RE were injected into the nuclei of single cells using a FluidFM Nanosyringe. To monitor injection efficiency, 12.5% of the gRNA was labeled with ATTO 550 dye. At 24 h postinjection, the cells were imaged using the FluidFM OMNIUM Platform for injection efficiency and survival by imaging for GFP fluorescence.

RPE-1 and U2OS cells were expanded and genotyped by PCR. Sequences of the gRNAs and the genotyping primers are listed in Supplementary Table 10.

### Reporter gene assays

Convergent promoters of the p53 target genes *BAX*, *PTP4A1* and *CCNG1* were amplified from MCF-7 genomic DNA and cloned into a pGL4.10 luciferase reporter vector (Promega) using KpnI and HindIII restriction sites. Upstream and downstream promoters including predicted p53REs^60^ were removed using alternative cloning primers (Supplementary Table 10). Dual-luciferase assays in U2OS cells were performed as described previously^61^.

The *GADD45A* gene was cloned into the pNL1.1 nanoluciferase reporter vector (Promega), resulting in a GADD45A-nLUC fusion system, as described previously^62^. One construct contained the wild-type *GADD45A* gene locus, while the downstream antisense promoter was deleted in a second construct. U2OS cells were transfected with 250 ng of NanoLuciferase reporter plasmid (pNL1.1) and 50 ng of Firefly luciferase plasmid (Promega, cat. no. pGL4.53). After overnight culture, cells were treated with Nutlin-3a or DMSO control for 24 h. Cells were collected and luciferase activity was measured using the Nano-Glo Dual-Luciferase Assay Kit (Promega) and a GloMax 20/20 luminometer (Promega) following the manufacturerʼs instructions.

### Reverse transcription semi-quantitative real-time PCR

Total cellular RNA was extracted using the innuPREP RNA Mini Kit (Analytik Jena) following the manufacturerʼs protocol. One-step reverse transcription and real-time semi-quantitative PCR (RT-qPCR) was performed with a Quantstudio v.5 using Power SYBR Green RNA-to-CT 1-Step Kit (ThermoFisher Scientific) following the manufacturerʼs protocol. Primer sequences are listed in Supplementary Table 10.

### Illumina sequencing and data preprocessing

MCF-7, RPE-1 and U2OS cells were treated with Nutlin-3a to activate p53 signaling or with the DMSO solvent to serve as a negative control with four (MCF-7) or three (U2OS and RPE-1) biological replicates. Total RNA was extracted using the RNeasy Plus Mini Kit (Qiagen) following the manufacturer’s protocol. Sequencing of RNA samples was performed using Illumina’s next-generation sequencing methodology^63^. In detail, total RNA was quantified and quality checked using a 2100 Bioanalyzer in combination with RNA 6000 assay or a 4200 Tapestation instrument in combination with RNA ScreenTape (Agilent Technologies). CAGE–seq^64^ libraries were prepared from 5,000 ng of total RNA using the CAGE Preparation Kit (Kabushiki Kaisha DNAFORM) following the manufacturer’s instructions. QuantSeq^51^ libraries were prepared from 500 ng of total RNA using the QuantSeq 3′ mRNA-Seq Library Prep Kit REV (Lexogen) following the manufacturer’s instructions. RNA-seq libraries were prepared from 800 ng of total RNA using NEBNext Ultra II Directional RNA Library Preparation Kit in combination with NEBNext Poly(A) mRNA Magnetic Isolation Module and NEBNext Multiplex Oligos for Illumina (Index Primers Set 1/2/3/4) following the manufacturer’s instructions (New England Biolabs) including size-selection at around 500 bp. Quantification and quality check of libraries was done using a 2100 Bioanalyzer instrument and DNA 7500 kit or a 4200 Tapestation instrument and a DNA 1000 kit (Agilent Technologies). Libraries were pooled and sequenced on a HiSeq 2500 using 50 cycle high-output reagents, a NextSeq 500 v.2 300 cycles run, a NextSeq 500 using 75 cycle high-output v.2.5 reagents or a NovaSeq 6000 SP 100 cycle v.1.5 run.

Inferred from FastQC (https://www.bioinformatics.babraham.ac.uk/projects/fastqc/) v.0.11.9 reports, we used Trimmomatic^65^ v.0.39 (5-nt sliding window approach, mean quality cutoff 22) for read quality trimming. Illumina universal adapter as well as mono- and dinucleotide content was clipped using Cutadapt v.2.10 (ref. ^66^). Potential sequencing errors were detected and corrected using Rcorrector v.1.0.4 (ref. ^67^). Ribosomal RNA (rRNA) transcripts were artificially depleted in RNA-seq data by read alignment against rRNA databases through SortMeRNA v.2.1 (ref. ^68^). In addition, Cutadapt was applied on CAGE–seq data using the nonshifting 5′ adapter ‘XG’ to clip a leading guanine and thus to correct for CAGE–seq’s typical 5′-end guanine addition bias. The preprocessed data was aligned to the reference genome hg38, retrieved along with its gene annotation from Ensembl v102 (ref. ^69^), using the mapping software segemehl^70,71^ v.0.3.4 with adjusted accuracy (95%) and split-read option enabled (RNA-seq) or disabled (CAGE–seq and QuantSeq). Mappings were filtered for uniqueness and properly aligned mate pairs (paired-end data only) with Samtools v.1.12 (ref. ^72^).

For visualization using the University of California Santa Cruz (UCSC) genome browser^73^, CAGE–seq and RNA-seq data were adjusted for library size differences and are displayed as normalized read counts.

### ATAC–seq and data processing

Biological quadruplets of MCF-7 cells treated with Nutlin-3a and DMSO control were utilized, with ATAC following the Omni-ATAC protocol^74^. Briefly, dead cells were removed using Annexin V magnetic beads (Miltenyi Biotec) and the remaining cells were treated with 200 U ml^−1^ DNase. Subsequently, 50,000 cells were pelleted at 500*g* at 4 °C and resuspended in lysis buffer (10 mM Tris-HCl, 10 mM NaCl, 3 mM MgCl_2_, 0.1% NP40, 0.1% Tween-20, 0.01% digitonin). After 3 min, the lysis buffer was washed out with 1 ml resuspension buffer (10 mM Tris-HCl, 10 mM NaCl, 3 mM MgCl_2_, 0.1% Tween-20), cells were pelleted (500*g*, 4 °C, 10 min) and resuspended in 50 µl transposition mixture (25 µl 2× TD buffer (Illumina), 2.5 µl TD enzyme (Illumina), 16.5 µl PBS, 0.5 µl 1% digitonin, 0.5 µl 10% Tween-20, 5 µl H_2_O). After incubation (37 °C, 1,000 rpm, 30 min), DNA was purified and Illumina adapters were added. The libraries were pooled and sequenced on a HiSeq 2500 using a 100 cycle (50-bp paired-end) rapid run.

We used FastQC, Timmomatic, Cutadapt and Rcorrector as described above. The reads were aligned to hg38 using segemehl (split-read option disabled). Mappings were filtered for uniqueness and properly aligned mate pairs with Samtools. The uniquely mapped reads in convergent promoters were counted with featureCounts v.2.0.3 (ref. ^75^) and differences (log_2_ fold change (FC)) between Nutlin-3a and DMSO control samples were calculated with DESeq2 v.1.34.0. We used DANPOS v.3.0.0 (ref. ^76^) to obtain normalized read fractions from nucleosome-free (fragments < 100 bp) and mononucleosome (180–240 bp) regions, as described previously^77^.

### GRO-seq analysis

GRO-seq data from MCF-7 cells treated with Nutlin-3a and DMSO control were retrieved from GSE86165 (ref. ^78^) and GSE53499(ref. ^79^). The data were analyzed using FastQC, Timmomatic, Cutadapt, Rcorrector and SortMeRNA as described above. Reads were aligned to hg38 using segemehl (split-read option disabled). The uniquely mapped reads in convergent promoters were counted with featureCounts v.2.0.3 and differences (log_2_FC) between Nutlin-3a and DMSO control samples were calculated using DESeq2 v.1.34.0.

### Nanopore sequencing and data processing

MCF-7 cells were treated with Nutlin-3a to activate p53 signaling or with the DMSO solvent to serve as a negative control. Total RNA was extracted using the innuPREP RNA Mini Kit (Analytik Jena) following the manufacturer’s protocol. Oxford Nanopore library preparation protocol SQK-PCB109 was followed using 50 ng of total RNA as input. The samples were barcoded according to protocol with 16 PCR cycles. Libraries were run for 72 h according to the guidelines of the manufacturer using Mk1B-MinION sequencer and R9.4.1 flow cells (FLO-MIN106D; Oxford Nanopore Technologies). Sequencing run monitoring and real-time data acquisition were performed using the MinKNOW software suite v.22.03.6 (Oxford Nanopore Technologies). Base calling was performed using guppy v.6.0.7 with the fast model (Oxford Nanopore Technologies). After identification, orientation and trimming of full-length cDNA reads using pychopper v.2.7.2 (Oxford Nanopore Technologies), reads were aligned to hg38 by minimap2 v.2.24-r1122 in spliced long-read mode and with disabled secondary alignments^80^.

### Identification of TSSs and TTSs

According to the library preparation methods, aligned CAGE–seq and QuantSeq data were split into strand-specific subsets using Samtools to subsequently call strand-specific peaks using PEAKachu v.0.2.0 (ref. ^81^) in adaptive mode, given all replicates. Peaks within a distance of 50 bp were merged with BEDTools v.2.30.0 (ref. ^82^). CAGE–seq-detected TSS (CTSS) and QuantSeq-detected TTS (QTTS) were obtained through BEDTools genomecov with the 5′-end (CTSS) or 3′-end (QTTS) coverage parameter, followed by BEDOPS^83^ v.2.4.32 max-element tool to determine TSSs and TTSs by local maxima, respectively.

### Identification of convergent promoters

To obtain a core set of convergent promoters composed of four TSSs as shown in Fig. 1d, divergent TSSs (−strand TSS followed by *+*strand TSS) were paired within 400 bp windows—a threshold established previously^40^. Afterwards, divergent TSS pairs were paired within a range of 2,500 bp, as indicated by distance density analysis (Extended Data Fig. 1c) and filtered for overlap with a GENCODE-annotated TSS on the same strand. Therefore, convergent promoter strandness and gene association were prior inferred from the most strongly expressed TSS, defined as TSS2.

To extend the set of convergent promoters, convergent TSSs (+strand TSS followed by −strand TSS) were paired within the 2,500 bp region and filtered by overlap a TSS annotated in GENCODE v.36/Ensembl v.102. The overlapping TSS was defined as host TSS. In case of ambiguousness, TSS expression defined the convergent promoter orientation.

### Identification of daRNAs

daRNAs were annotated when no gene start annotation was available at the daTSS starting from convergent promoter in MCF-7. Transcript bodies were identified using a sliding window of length 100 and a coverage threshold of ten reads on RNA-seq data. Elongation was terminated upon reaching a known TSS complemented by a CAGE–seq peak on the same strand; daRNA transcripts and their 3′-end were annotated by QuantSeq-derived QTTS and ranked according to its expression. The dominant daRNA was defined as the transcript with the strongest QTTS and daTSS expression.

### Differential expression analysis

RNA-seq read quantification was performed on exon level while CAGE–seq and QuantSeq read quantification was performed on peak level using featureCounts v.2.0.3 (ref. ^75^), parametrized according to the experiments library strandness and subsequently tested for differential expression. Differential gene expression and its statistical significance was identified using DESeq2 v.1.34.0 (ref. ^84^) and adjusted for multiple testing via the Benjamini–Hochberg procedure.

### Single-molecule fluorescent in situ hybridization

Stellaris probe sets for single-molecule fluorescent in situ hybridization (smFISH) (Biosearch Technologies) were custom-designed for targeting the first intron of both *FAS* and *ACTA2*. Each probe set was comprised of 48 5′–3′ complementary oligonucleotides 22 nt in length, masking level 5 and a minimum spacing length of 2 nt. The *FAS* probe was labeled with CAL Fluor Red 610 and the *ACTA2* probe with Quasar 610 dye. MCF-7 cells were grown for 2 days on 18-mm uncoated coverglasses (thickness 1). After treatment with 10 μM Nutlin-3a (MedChemExpress), cells were washed with sterile ice-cold PBS at indicated timepoints, fixed with 2% paraformaldehyde (PFA) (electron microscopy grade) for 10 min at room temperature and permeabilized with 70% ethanol at 4 °C overnight. A custom probe set was hybridized for 16 h at a final concentration of 0.1 μM according to the Stellaris RNA FISH manufacturer. Afterwards, cells were washed and incubated with Hoechst 33342 for 10 min at room temperature for nuclear staining. Coverslips were then mounted on Prolong Gold Antifade (Molecular Probes, Life Technologies). Cells were imaged on a Nikon ECLIPSE Ti-E inverted fluorescence microscope with an F-mount camera DS-Qi2 equipped with CMOS image sensor. A ×60x plan apo objective (NA 1.4) and appropriate filter sets were used: (Hoechst: 387/11-nm excitation (EX), 409-nm dichroic beam splitter (BS), 447/60-nm emission (EM); CAL Fluor Red 610: 580/25-nm EM, 600-nm BS, 625-nm EX; Quasar 670: 640/30-nm EX, 660-nm BS, 690/50-nm EM). Images were acquired as multipoint of 21 *z*-stacks of each group of cells in a field of view with 300-nm step-width using Nikon Elements software (Nikon Instruments). After extraction of multicolor *z*-stacks and maximum intensity *Z*-projection, fluorescent intensity profiles of single regions of interest were plotted in ImageJ/Fiji.

### Tau index analysis

Tissue-specific TSS activity has been assessed through the Tau index^85^ using FANTOM5 CAGE–seq data^86^. The hg19 peaks were converted to hg38 using UCSC liftover^87^. The Tau index was calculated with tispec v.0.99.0 (https://github.com/BioinfGuru/tispec).

### Statistics and reproducibility

No statistical method was used to predetermine sample size but our sample sizes are similar to those reported in previous publications^18,40,45^. No data were excluded from the analyses. The experiments were not randomized. Investigators were not blinded to allocation during experiments and outcome assessment. Data distribution was not formally tested/analyzed for all correlation analyses. Thus, the nonparametric Spearman rank correlation with two-sided significance was calculated. Data distributions were also not formally tested/analyzed for all read count analyses and hence evaluated with the nonparametric unpaired, two-sided Wilcoxon rank sum test (Fig. 6e and Extended Data Fig. 8c,d). Normal distribution was assumed but not formally tested for dual-luciferase assays (Fig. 4 and Extended Data Fig. 7d) and RT-qPCR analysis (Extended Data Fig. 10d). Here, an unpaired, two-tailed *t*-test was used.

### Reporting summary

Further information on research design is available in the Nature Portfolio Reporting Summary linked to this article.

## Online content

Any methods, additional references, Nature Portfolio reporting summaries, source data, extended data, supplementary information, acknowledgements, peer review information; details of author contributions and competing interests; and statements of data and code availability are available at 10.1038/s41588-024-02025-w.

## Supplementary information

Supplementary Information Supplementary Discussion and Supplementary Table Legends.

Reporting Summary

Supplementary Tables 1–10 Supplementary Table 1. Identification of a core set of convergent promoters in MCF-7 cells. We identified a core set of convergent promoters following the flowchart of Extended Data Fig. 1a. The table contains annotations and differential expression information. Differential gene expression and its statistical significance was identified using DESeq2 v.1.34.0 and adjusted for multiple testing via the Benjamini–Hochberg procedure. Supplementary Table 2. Identification of a core set of convergent promoters in RPE-1 cells. We identified a core set of convergent promoters following the flowchart of Extended Data Fig. 1a. The table contains annotations and differential expression information. Differential gene expression and its statistical significance was identified using DESeq2 v.1.34.0 and adjusted for multiple testing via the Benjamini–Hochberg procedure. Supplementary Table 3. Identification of a core set of convergent promoters in U2OS cells. We identified a core set of convergent promoters following the flowchart of Extended Data Fig. 1a. The table contains annotations and differential expression information. Differential gene expression and its statistical significance was identified using DESeq2 v.1.34.0 and adjusted for multiple testing via the Benjamini–Hochberg procedure. Supplementary Table 4. Identification of a core set of convergent promoters in the joint data. We identified a core set of convergent promoters following the flowchart of Extended Data Fig. 1a. The Table contains annotations and differential expression information. Differential gene expression and its statistical significance was identified using DESeq2 v.1.34.0 and adjusted for multiple testing via the Benjamini–Hochberg procedure. Supplementary Table 5. Identification of an extended set of convergent promoters in MCF-7 cells. We identified an extended set of convergent promoters by directly pairing convergent CAGE peaks using the 2.5 kb threshold we established. Subsequently, we selected all pairs overlapping with a TSS of a GENCODE-annotated gene. The dominant TSS was defined as the hostTSS. The table contains annotations and differential expression information. Differential gene expression and its statistical significance was identified using DESeq2 v.1.34.0 and adjusted for multiple testing via the Benjamini–Hochberg procedure. Supplementary Table 6. Identification of an extended set of convergent promoters in RPE-1 cells. We identified an extended set of convergent promoters by directly pairing convergent CAGE peaks using the 2.5 kb threshold we established. Subsequently, we selected all pairs overlapping with a TSS of a GENCODE-annotated gene. The dominant TSS was defined as the hostTSS. The table contains annotations and differential expression information. Differential gene expression and its statistical significance was identified using DESeq2 v.1.34.0 and adjusted for multiple testing via the Benjamini–Hochberg procedure. Supplementary Table 7. Identification of an extended set of convergent promoters in U2OS cells. We identified an extended set of convergent promoters by directly pairing convergent CAGE peaks using the 2.5 kb threshold we established. Subsequently, we selected all pairs overlapping with a TSS of a GENCODE-annotated gene. The dominant TSS was defined as the hostTSS. The table contains annotations and differential expression information. Differential gene expression and its statistical significance was identified using DESeq2 v.1.34.0 and adjusted for multiple testing via the Benjamini–Hochberg procedure. Supplementary Table 8. Identification of an extended set of convergent promoters in the joint data. We identified an extended set of convergent promoters by directly pairing convergent CAGE peaks using the 2.5 kb threshold we established. Subsequently, we selected all pairs overlapping with a TSS of a GENCODE-annotated gene. The dominant TSS was defined as the hostTSS. The table contains annotations and differential expression information. Differential gene expression and its statistical significance was identified using DESeq2 v.1.34.0 and adjusted for multiple testing via the Benjamini–Hochberg procedure. Supplementary Table 9. Host gene/daRNA pairs in MCF-7 cells. The table contains host gene/daRNA pairs regulated by convergent promoters including novel daRNAs that we uncovered by combining CAGE–seq, RNA-seq and QuantSeq data. Detailed information on the annotation of dominant daRNAs is displayed. Supplementary Table 10. Oligonucleotides. The table contains oligonucleotides that have been used, including primers and guide RNAs.

## Source data

Source Data Fig. 1 Source Data.

Source Data Fig. 2 Source Data.

Source Data Fig. 3 Source Data.

Source Data Fig. 4 Source Data.

Source Data Fig. 5 Source Data.

Source Data Fig. 6 Source Data.

Source Data Fig. 7 Source Data.

Source Data Extended Data Fig. 1 Source Data.

Source Data Extended Data Fig. 2 Source Data.

Source Data Extended Data Fig. 3 Source Data.

Source Data Extended Data Fig. 4 Source Data.

Source Data Extended Data Fig. 5 Source Data.

Source Data Extended Data Fig. 7 Source Data.

Source Data Extended Data Fig. 8 Source Data.

Source Data Extended Data Fig. 9 Source Data.

Source Data Extended Data Fig. 10 Source Data.

## Data Availability

The hg38 genome and its annotation were obtained from ENSEMBL v.102 (ref. ^69^). Transcription factor binding data on p53, E2F4 and RFX7 are available through www.targetgenereg.org (ref. ^57^). GRO-seq data from MCF-7 cells are publicly available through GSE86165 (ref. ^78^) and GSE53499 (ref. ^79^). RNA-seq data from RFX7-depleted U2OS cells are publicly available through GSE162163 (ref. ^45^). Epigenetic data are publicly available through ENCODE^56^: ATAC–seq (ENCFF782BVX), H2AFZ (ENCFF740HVA), H3K4me1 (ENCFF763NCP), H3K4me3 (ENCFF163MXP), H3K9ac (ENCFF327XJC), H3K27ac (ENCFF138YNG), H3K27me3 (ENCFF163QKN), H3K36me3 (ENCFF910BRP), H3K79me2 (ENCFF826OGB), H4K20me1 (ENCFF366GLZ) and Pol II (ENCFF827YIP). Hg38 CpG island data are available through the UCSC genome browser (http://hgdownload.cse.ucsc.edu/goldenpath/hg38/database/cpgIslandExt.txt.gz)^88^. G4 ChIP–seq data from U2OS cells are publicly available through GSE162299 (ref. ^89^). DRIP–seq (R-loop) data from MCF-7 cells are publicly available through GSE81851 (ref. ^90^) and GSE98886 (ref. ^91^) and from U2OS cells through GSE115957 (ref. ^92^) and GSE155865 (ref. ^93^). RNA-seq data from estradiol (E2)-treated MCF-7 cells are publicly available through GSE117942 (ref. ^94^) and GSE173976 (ref. ^95^). In addition, our sequencing data are accessible through GEO^96^. CAGE–seq data are available through GSE223512. RNA-seq data are available through GSE216721 (MCF-7), GSE173483 (U2OS) and GSE216720 (RPE-1). QuantSeq data are available through GSE223513. Nanopore sequencing data are available through GSE226080. ATAC–seq data from Nutlin-3a and DMSO control-treated MCF-7 cells are available through GSE250017. Source data are provided with this paper.

## Code availability

The code used for data analysis is available via Zenodo at 10.5281/zenodo.14011612 (ref. ^97^).
