# Supplementary material for: Gene regulation by convergent promoters
Source: Nat Genet. 2025 Jan 6;57(1):206–17. doi: 10.1038/s41588-024-02025-w (PMC11735407; doi:10.1038/s41588-024-02025-w)
Supplement: Supplementary file 1 — Supplementary Discussion and Supplementary Table Legends. [file 41588_2024_2025_MOESM1_ESM.pdf]

nature genetics

Supplementary information https://doi.org/10.1038/s41588-024-02025-w

Gene regulation by convergent promoters

In the format provided by the authors and unedited

Supplementary Discussion

Over the past decade, traditional classifications of promoters and enhancers became blurred. Epigenetic signals, the initiation of a stable downstream transcription unit, and GC content are major hallmarks for distinguishing between promoters and enhancers [1–3]. Downstream antisense promoters share many characteristics with intragenic enhancers. However, downstream antisense promoters frequently overlap CGIs (**Fig. 6b**) and show histone marks indicative of active promoters and not enhancers (**Fig. 6c**). Furthermore, our data show that the downstream antisense promoters initiate stable daRNAs with an average length of several kilobases and well-defined transcription start and termination sites (**Fig. 7a-d**). Thus, our data provides evidence that the described downstream elements may be best characterized as promoters. Additionally, it has been suggested that intragenic enhancer activity interferes with host gene transcription [4]. Here, we found that intragenic promoters, at least as part of a cocoProm, do not prohibit the expression of their host genes, revealing limitations of the proposed model of intragenic enhancer logic. While our data cannot resolve the coordinating mechanism of convergent transcription, we provide evidence that the presence of juxtaposed Pol II promoters does not impair transcription in any direction. Recently, it has been shown that Pol II can, at least in part, rewrap nucleosomes to retain chromatin structure [5]. However, from a biochemical perspective, we find it difficult to imagine that the entire promoter, in particular transcription factor binding, can be preserved during Pol II passage. Instead, we find it intriguing to hypothesize that condensates [6] can form at cocoProms, perhaps supported by CGIs, G4 structures, and R-loops, that keep the critical factors in close proximity to enable a rapid reassembly and subsequent Pol II loading after antisense Pol II passage. Such a rapid promoter reassembly model would explain why any transcription interference caused by Pol II promoter passage is masked when a locus undergoes alternating convergent transcription by Pol II complexes. Alternating convergent transcription is also supported by GRO-seq data that indicate Pol II pausing between TSS#2 and TSS#4 in sense direction, while Pol II initiated from TSS#3 pauses just behind TSS#1 in antisense (**Extended Data Fig. 8a**). In such a constellation, Pol II pause release would not lead to a collision between the converging Pol IIs. Alternatively, it may turn out that converging Pol II complexes in metazoans can actually bypass each other.

Convergent promoters share many characteristics with other classes of promoters, but they also have distinct differences. First, convergent promoters initiate bi-directional transcription, which is the key feature of bi-directional promoters. While classical bi-directional promoters initiate divergent transcription from a single nucleosome-depleted promoter region [7], convergent promoter constellations instead use two distinct nucleosome-depleted promoter regions to initiate convergent transcription. Second, the TSS#4 of the prototypical convergent

promoter constellation (**Fig. 1d**) often overlaps with a known alternative TSS of the host gene (**Extended Data Fig. 2a**), and in these cases, the downstream antisense promoter also represents an alternative host gene promoter. However, the majority of TSS#4s do not overlap with an alternative host gene TSS, and many convergent promoters do not contain a detectable TSS#4 (see 'extended set'). Similarly, the vast majority of protein-coding genes have multiple known TSSs, but only a minority are regulated by convergent promoters. Third, convergent promoters are highly enriched for CGIs, and thus most convergent promoters are CGI promoters. While about 70 % of all promoters are CGI promoters [8], only about a quarter of all promoters are juxtaposed to another promoter with convergent transcription between them. Thus, convergent promoters can be distinguished from other well-established promoter classes with which they share key characteristics.

Supplementary Discussion References

1. Kim, T.-K. & Shiekhattar, R. Architectural and Functional Commonalities between Enhancers and Promoters. *Cell* **162**, 948–959 (2015).
2. Haberle, V. & Stark, A. Eukaryotic core promoters and the functional basis of transcription initiation. *Nat. Rev. Mol. Cell Biol.* **19**, 621–637 (2018).
3. Andersson, R. & Sandelin, A. Determinants of enhancer and promoter activities of regulatory elements. *Nat. Rev. Genet.* **21**, 71–87 (2020).
4. Cinghu, S. *et al.* Intragenic Enhancers Attenuate Host Gene Expression. *Mol. Cell* **68**, 104-117.e6 (2017).
5. Filipovski, M., Soffers, J. H. M., Vos, S. M. & Farnung, L. Structural basis of nucleosome retention during transcription elongation. *Science* **376**, 1313–1316 (2022).
6. Cramer, P. Organization and regulation of gene transcription. *Nature* **573**, 45–54 (2019).
7. Wei, W., Pelechano, V., Järvelin, A. I. & Steinmetz, L. M. Functional consequences of bidirectional promoters. *Trends Genet.* **27**, 267–276 (2011).
8. Deaton, A. M. & Bird, A. CpG islands and the regulation of transcription. *Genes Dev.* **25**, 1010–1022 (2011).

Supplementary Tables Legends

Supplementary Table 1. Identification of a core set of convergent promoters in MCF-7 cells. We identified a core set of convergent promoters following the flow chart of Extended Data Fig. 1a. The Table contains annotations and differential expression information. Differential gene expression and its statistical significance was identified using DESeq2 v1.34.0 and adjusted for multiple testing via the Benjamini-Hochberg procedure.

Supplementary Table 2. Identification of a core set of convergent promoters in RPE-1 cells. We identified a core set of convergent promoters following the flow chart of Extended Data Fig. 1a. The Table contains annotations and differential expression information. Differential gene expression and its statistical significance was identified using DESeq2 v1.34.0 and adjusted for multiple testing via the Benjamini-Hochberg procedure.

Supplementary Table 3. Identification of a core set of convergent promoters in U2OS cells. We identified a core set of convergent promoters following the flow chart of Extended Data Fig. 1a. The Table contains annotations and differential expression information. Differential gene expression and its statistical significance was identified using DESeq2 v1.34.0 and adjusted for multiple testing via the Benjamini-Hochberg procedure.

Supplementary Table 4. Identification of a core set of convergent promoters in the joint data. We identified a core set of convergent promoters following the flow chart of Extended Data Fig. 1a. The Table contains annotations and differential expression information. Differential gene expression and its statistical significance was identified using DESeq2 v1.34.0 and adjusted for multiple testing via the Benjamini-Hochberg procedure.

Supplementary Table 5. Identification of an extended set of convergent promoters in MCF-7 cells. We identified an extended set of convergent promoters by directly pairing convergent CAGE peaks using the 2.5 kb threshold we established. Subsequently, we selected all pairs overlapping with a TSS of a GENCODE-annotated gene. The dominant TSS was defined as the hostTSS. The Table contains annotations and differential expression information. Differential gene expression and its statistical significance was identified using DESeq2 v1.34.0 and adjusted for multiple testing via the Benjamini-Hochberg procedure.

Supplementary Table 6. Identification of an extended set of convergent promoters in RPE-1 cells. We identified an extended set of convergent promoters by directly pairing convergent CAGE peaks using the 2.5 kb threshold we established. Subsequently, we

selected all pairs overlapping with a TSS of a GENCODE-annotated gene. The dominant TSS was defined as the hostTSS. The Table contains annotations and differential expression information. Differential gene expression and its statistical significance was identified using DESeq2 v1.34.0 and adjusted for multiple testing via the Benjamini-Hochberg procedure.

Supplementary Table 7. Identification of an extended set of convergent promoters in U2OS cells. We identified an extended set of convergent promoters by directly pairing convergent CAGE peaks using the 2.5 kb threshold we established. Subsequently, we selected all pairs overlapping with a TSS of a GENCODE-annotated gene. The dominant TSS was defined as the hostTSS. The Table contains annotations and differential expression information. Differential gene expression and its statistical significance was identified using DESeq2 v1.34.0 and adjusted for multiple testing via the Benjamini-Hochberg procedure.

Supplementary Table 8. Identification of an extended set of convergent promoters in the joint data. We identified an extended set of convergent promoters by directly pairing convergent CAGE peaks using the 2.5 kb threshold we established. Subsequently, we selected all pairs overlapping with a TSS of a GENCODE-annotated gene. The dominant TSS was defined as the hostTSS. The Table contains annotations and differential expression information. Differential gene expression and its statistical significance was identified using DESeq2 v1.34.0 and adjusted for multiple testing via the Benjamini-Hochberg procedure.

Supplementary Table 9. Host gene / daRNA pairs in MCF-7 cells. The table contains host gene / daRNA pairs regulated by convergent promoters including novel daRNAs that we uncovered by combining CAGE-seq, RNA-seq, and QuantSeq data. Detailled information on the annotation of dominant daRNAs is displayed.

Supplementary Table 10. Oligonucleotides. The table contains oligonucleotides that have been used. Including primers and guide RNAs.
