# Supplementary material for: Gene regulation by convergent promoters
Source: Nat Genet. 2025 Jan 6;57(1):206–17. doi: 10.1038/s41588-024-02025-w (PMC11735407; doi:10.1038/s41588-024-02025-w)
Supplement: Supplementary file 2 — Reporting Summary [file 41588_2024_2025_MOESM2_ESM.pdf]

nature portfolio

Corresponding author(s): Steve Hoffmann and Martin Fischer

Last updated by author(s): Sep 17, 2024

Reporting Summary

Nature Portfolio wishes to improve the reproducibility of the work that we publish. This form provides structure for consistency and transparency in reporting. For further information on Nature Portfolio policies, see our Editorial Policies and the Editorial Policy Checklist.

Statistics

For all statistical analyses, confirm that the following items are present in the figure legend, table legend, main text, or Methods section.

n/a	Confirmed	
☐	☒	The exact sample size (*n*) for each experimental group/condition, given as a discrete number and unit of measurement
☐	☒	A statement on whether measurements were taken from distinct samples or whether the same sample was measured repeatedly
☐	☒	The statistical test(s) used AND whether they are one- or two-sided<br>*Only common tests should be described solely by name; describe more complex techniques in the Methods section.*
☒	☐	A description of all covariates tested
☐	☒	A description of any assumptions or corrections, such as tests of normality and adjustment for multiple comparisons
☐	☒	A full description of the statistical parameters including central tendency (e.g. means) or other basic estimates (e.g. regression coefficient) AND variation (e.g. standard deviation) or associated estimates of uncertainty (e.g. confidence intervals)
☐	☒	For null hypothesis testing, the test statistic (e.g. *F*, *t*, *r*) with confidence intervals, effect sizes, degrees of freedom and *P* value noted<br>*Give P values as exact values whenever suitable.*
☒	☐	For Bayesian analysis, information on the choice of priors and Markov chain Monte Carlo settings
☒	☐	For hierarchical and complex designs, identification of the appropriate level for tests and full reporting of outcomes
☐	☒	Estimates of effect sizes (e.g. Cohen's *d*, Pearson's *r*), indicating how they were calculated

Our web collection on statistics for biologists contains articles on many of the points above.

Software and code

Policy information about availability of computer code

Data collection	Thermo Fisher Scientific Design and Analysis Software v1.5.1<br>Illumina bcl2FastQ<br>Nikon Elements software
Data analysis	FastQC v0.11.9<br>Trimmomatic v0.39<br>Cutadapt v2.10<br>Rcorrector v1.0.4<br>SortMeRNA v2.1<br>segemehl v0.3.4<br>Samtools v1.12<br>MinKNOW software suite v22.03.6<br>pychopper v2.7.2<br>minimap2 v2.24-r1122<br>PEAKachu v0.2.0<br>BEDTools v2.30.0<br>BEDOPS v2.4.32<br>featureCounts v2.0.3<br>DESeq2 v1.34.0<br>ImageJ/Fiji v1.53f<br>R v4.1.3

tidyverse v2.0.0
biomaRt v2.54.1
Python v3.9.9
pysam v0.18.0
pyranges v0.0.117
deeptools v3.5.0
genomation v1.30.0
ggrastr v1.0.2
fitdistrplus v1.1-11
GenomicRanges v1.50.2
Repitools v1.44.0
rstatix v0.7.2
ggpmisc v0.5.4-1
ggpubr v0.6.0
scales v1.2.1
plyranges v1.18.0
UCSC liftOver
tispec v0.99.0
featureCounts v2.0.3
DANPOS v3.0.0
guppy v6.0.7

For manuscripts utilizing custom algorithms or software that are central to the research but not yet described in published literature, software must be made available to editors and reviewers. We strongly encourage code deposition in a community repository (e.g. GitHub). See the Nature Portfolio guidelines for submitting code & software for further information.

Data

Policy information about availability of data

All manuscripts must include a data availability statement. This statement should provide the following information, where applicable:

- Accession codes, unique identifiers, or web links for publicly available datasets
- A description of any restrictions on data availability
- For clinical datasets or third party data, please ensure that the statement adheres to our policy

Data Availability
The hg38 genome and its annotation were obtained from ENSEMBL v102 69. Transcription factor binding data on p53, E2F4, and RFX7 are available through www.targetgenereg.org 57. GRO-seq data from MCF-7 cells are publicly available through GSE86165 78 and GSE53499 79. RNA-seq data from RFX7 depleted U2OS cells are publicly available through GSE162163 45. Epigenetic data are publicly available through ENCODE 56: ATAC-seq (ENCFF782BVX), H2AFZ (ENCFF740HVA), H3K4me1 (ENCFF763NCP), H3K4me3 (ENCFF163MXP), H3K9ac (ENCFF327XJC), H3K27ac (ENCFF138YNG), H3K27me3 (ENCFF163QKN), H3K36me3 (ENCFF910BRP), H3K79me2 (ENCFF826OGB), H4K20me1 (ENCFF366GLZ), and Pol II (ENCFF827YIP). Hg38 CpG island data are available through the UCSC genome browser (http://hgdownload.cse.ucsc.edu/goldenpath/hg38/database/cpgIslandExt.txt.gz) 88. G4 ChIP-seq data from U2OS cells are publicly available through GSE162299 89. DRIP-seq (R-loop) data from MCF-7 cells are publicly available through GSE81851 90 and GSE98886 91 and from U2OS cells through GSE115957 92 and GSE155865 93. RNA-seq data from estradiol (E2)-treated MCF-7 cells are publicly available through GSE117942 94 and GSE173976 95.
In addition, our sequencing data are accessible through GEO 96. CAGE-seq data are available through GSE223512. RNA-seq data are available through GSE216721 (MCF-7), GSE173483 (U2OS), and GSE216720 (RPE-1). QuantSeq data are available through GSE223513. Nanopore sequencing data are available through GSE226080. ATAC-seq data from Nutlin-3a and DMSO control-treated MCF-7 cells are available through GSE250017.

Research involving human participants, their data, or biological material

Policy information about studies with human participants or human data. See also policy information about sex, gender (identity/presentation), and sexual orientation and race, ethnicity and racism.

Reporting on sex and gender	n/a
Reporting on race, ethnicity, or other socially relevant groupings	n/a
Population characteristics	n/a
Recruitment	n/a
Ethics oversight	n/a

Note that full information on the approval of the study protocol must also be provided in the manuscript.

Field-specific reporting

Please select the one below that is the best fit for your research. If you are not sure, read the appropriate sections before making your selection.

☒ Life sciences ☐ Behavioural & social sciences ☐ Ecological, evolutionary & environmental sciences

For a reference copy of the document with all sections, see nature.com/documents/nr-reporting-summary-flat.pdf

Life sciences study design

All studies must disclose on these points even when the disclosure is negative.

Sample size	No statistical method was used to predetermine sample size but our sample sizes are similar to those reported in previous publications 18,40,45.
Data exclusions	No data were excluded from the analyses.
Replication	Experiments were performed at least two times, additionally most findings were replicated in two or three cell line models. All findings were replicated successfully. The number of replicates is indicated in the respective Figure legend.
Randomization	The experiments in our study could not be randomized.
Blinding	Investigators were not blinded because conditions were required knowledge for carrying out the experiments.

Reporting for specific materials, systems and methods

We require information from authors about some types of materials, experimental systems and methods used in many studies. Here, indicate whether each material, system or method listed is relevant to your study. If you are not sure if a list item applies to your research, read the appropriate section before selecting a response.

Materials & experimental systems

n/a	Involved in the study	
☒	☐	Antibodies
☐	☒	Eukaryotic cell lines
☒	☐	Palaeontology and archaeology
☒	☐	Animals and other organisms
☒	☐	Clinical data
☒	☐	Dual use research of concern
☒	☐	Plants

Methods

n/a	Involved in the study	
☒	☐	ChIP-seq
☒	☐	Flow cytometry
☒	☐	MRI-based neuroimaging

Eukaryotic cell lines

Policy information about cell lines and Sex and Gender in Research

Cell line source(s)	MCF-7, U2OS, and RPE-1 were obtained from ATCC.
Authentication	Cell authentication was performed using morphological validation
Mycoplasma contamination	Cell lines were tested twice a year for Mycoplasma contamination using the LookOut Detection Kit (Sigma Aldrich, Darmstadt, Germany), and all tests were negative.
Commonly misidentified lines (See ICLAC register)	None of these cell lines were used.
